# Evaluating Phage Tail Fiber Receptor-Binding Proteins Using a Luminescent Flow-Through 96-Well Plate Assay

**DOI:** 10.3389/fmicb.2021.741304

**Published:** 2021-12-16

**Authors:** Emma L. Farquharson, Ashlyn Lightbown, Elsi Pulkkinen, Téa Russell, Brenda Werner, Sam R. Nugen

**Affiliations:** Nugen Research Laboratory, Department of Food Science, Cornell University, Ithaca, NY, United States

**Keywords:** tailed phages, NanoLuc luciferase, phage receptor binding proteins, ECOR Reference Library, phage adsorption, bacterial phage binding assay, bacteriophage T4, phage-host interaction

## Abstract

Phages have demonstrated significant potential as therapeutics in bacterial disease control and as diagnostics due to their targeted bacterial host range. Host range has typically been defined by plaque assays; an important technique for therapeutic development that relies on the ability of a phage to form a plaque upon a lawn of monoculture bacteria. Plaque assays cannot be used to evaluate a phage’s ability to recognize and adsorb to a bacterial strain of interest if the infection process is thwarted post-adsorption or is temporally delayed, and it cannot highlight which phages have the strongest adsorption characteristics. Other techniques, such as classic adsorption assays, are required to define a phage’s “adsorptive host range.” The issue shared amongst all adsorption assays, however, is that they rely on the use of a complete bacteriophage and thus inherently describe when all adsorption-specific machinery is working together to facilitate bacterial surface adsorption. These techniques cannot be used to examine individual interactions between a singular set of a phage’s adsorptive machinery (like long tail fibers, short tail fibers, tail spikes, etc.) and that protein’s targeted bacterial surface receptor. To address this gap in knowledge we have developed a high-throughput, filtration-based, bacterial binding assay that can evaluate the adsorptive capability of an individual set of a phage’s adsorption machinery. In this manuscript, we used a fusion protein comprised of an N-terminal bioluminescent tag translationally fused to T4’s long tail fiber binding tip (gp37) to evaluate and quantify gp37’s relative adsorptive strength against the *Escherichia coli* reference collection (ECOR) panel of 72 *Escherichia coli* isolates. Gp37 could adsorb to 61 of the 72 ECOR strains (85%) but coliphage T4 only formed plaques on 8 of the 72 strains (11%). Overlaying these two datasets, we were able to identify ECOR strains incompatible with T4 due to failed adsorption, and strains T4 can adsorb to but is thwarted in replication at a step post-adsorption. While this manuscript only demonstrates our assay’s ability to characterize adsorptive capabilities of phage tail fibers, our assay could feasibly be modified to evaluate other adsorption-specific phage proteins.

## Introduction

Bacteriophages (or “phages”) are the natural and ubiquitous viral predators of bacteria: relying on their bacterial host’s machinery and energy reserves in order to replicate (Kutter and Sulakvelidze, 2004). Predation starts with phage recognition of a bacterial species within a specific host range: a complex process that ultimately leads to infection and death of the bacterial cell and release of progeny phages (Hampton et al., 2020). The predatory nature of phages on bacteria, their ability to be genetically engineered using a wide-range of techniques, and their definable host range toward many pathogenic bacteria has resulted in interest of phage-based technologies as tools for therapeutics, diagnostics, biocontrol, and even vaccines. Research of phage therapy declined following the introduction of antibiotics in the late 1930s, but emergence of multi-drug resistant bacteria has led to renewed interest in the field. Phage therapy has only been approved for cases of compassionate use within the European Union and United States, but there are an increasing number of examples were phage therapy has allowed for the recovery of patients where all other treatment options failed (Schooley et al., 2017; LaVergne et al., 2018; Aslam et al., 2019a,b, 2020; Law et al., 2019). Beyond utilizing wild-type phages, a relatively recent phage therapy case demonstrated the first use of genetically engineered phages when a 15-year-old female with cystic fibrosis was successfully treated for a *Mycobacterium abscessus* infection (Dedrick et al., 2019). Genetic engineering of phages using techniques such as homologous recombination (Pines et al., 2015), BRED (Marinelli et al., 2008), CRISPR Cas9 (Tao et al., 2017; Duong et al., 2020a), *in vivo* yeast assembly (Ando et al., 2015), and *in vitro* Gibson assembly (Pulkkinen et al., 2019) has also allowed phages to serve as biological indicators of their host bacteria; enabling their use as biosensors for bacterial contaminants within food, water, and environmental samples.

For tailed phages, initial recognition of a permissive host is facilitated by specific interactions between phage tail fibers or tail spike proteins, and bacterial surface receptors (Letarov and Kulikov, 2017). While not all tailed phages are the same, many require bacterial adsorption using both long and short tail fibers before the phage can initiate genome translocation (Storms and Sauvageau, 2015): such is the case for the most well-known of all tailed phages, coliphage T4. The current paradigm for phage discovery involves isolation of phages from environmental or clinical samples; and while this step is necessary for basic research, the process is exhaustive and laborious. Advances in synthetic biology may soon allow a bottom-up approach for engineering custom phages or phage tail fibers, but for this to become reality we must first have a better understanding of phage-host interactions and especially mechanisms of tail fiber adsorption. Research which aims to deepen our understanding of phage-host interactions provides the key foundation from which all future technological developments for phage-based tools will derive. No matter the phage in question, however, adsorption to a permissive host is the first step of a successful infection and often serves as the initial predictor of a phage’s bacterial host range (d’Herelle and Smith, 1926; Clokie, 2009; Clokie and Kropinski, 2009; Letarov and Kulikov, 2017). Host range has typically been defined by plaque assays; an important technique for therapeutic development that relies on the ability of a phage to form a plaque upon a lawn of monoculture bacteria but that can only identify bacterial strains a phage is able to replicate within. Plaque assays are count individual plaque forming units (PFUs) formed after multiple rounds of phage infections within permissive hosts have occurred, but they cannot be used to solely evaluate a phage’s ability to recognize and adsorb to a bacterial strain of interest if the infection process is thwarted post-adsorption or is temporally delayed. It also cannot highlight which phages have the strongest adsorption characteristics, requiring other techniques such as classic adsorption assays to define a phage’s “adsorptive host range.”

One such technique is to compare plaque assay results to that of spot assay results and search for instances of abortive infections or “lysis from without”; a phenomenon where too many phages bind at one time to a single bacterial cell and cause drastic membrane destabilization and the collapse of the bacterium before a phage can complete its replication and release progeny (Abedon, 2011). While many other assays for defining phage adsorption already exist, these methods rely on the use of a complete phage and are inherently a description of all adsorption-specific machinery working together to facilitate bacterial surface adsorption. Unfortunately, none of these techniques can be used to investigate individual interactions between a singular set of a phage’s adsorptive machinery (i.e., long tail fibers, short tail fibers, tail spikes, etc.) and that protein’s targeted receptor.

To address this deficit, we developed a high-throughput, filtration-based, bacterial binding assay that evaluates the adsorptive capability of an individual set of phage adsorption machinery. We expressed luciferase-tagged (NanoLuc) T4 long tail fiber adhesin (gp37) proteins and challenged them against the *Escherichia coli* reference collection (ECOR) panel of 72 *Escherichia coli* isolates: representing the diversity of the species (Ochman and Selander, 1984). Being that T4 represents the archetype of tailed phages, a great deal of information already exists concerning its adsorptive capabilities and replicative functions (Wilson et al., 1970; Washizaki et al., 2016; Hyman and van Raaij, 2018; Kutter et al., 2018; Duong et al., 2020b), making it an ideal model organism. The assay relies on a 96-well filter plate to allow the potential of high throughput screening, as well as an N-terminal translational fusion to NanoLuc luciferase to serve as an indicator of gp37 adsorption. Adsorption results for gp37 were compared to the efficiency of plating (EOP) values from coliphage T4’s evaluation against the ECOR library. Gp37 could adsorb to 61 of the 72 ECOR strains (85%) but T4 only formed plaques on 8 of the strains (11%). The overlay of these datasets allowed for identification of both ECOR isolates incompatible with T4 due to failed adsorption, and isolates T4 can adsorb to but is thwarted at some point in replication post-adsorption. Especially when used alongside other well-known adsorption assays, classic spot tests, and standard full plate plaque assays, our high-throughput adsorption assay provides an important viewpoint from which researchers can form unique interpretations of adsorption data and possibly discover new fundamental concepts within the field of phage research. While this manuscript only demonstrates our assay’s ability to characterize the adsorptive capabilities of phage tail fibers, our assay could feasibly be modified to evaluate other adsorption-specific phage proteins. By creating a method in which individual interactions between a phage’s adsorptive machinery and targeted receptors can be examined, a new lens of understanding surrounding phage adsorption becomes possible.

## Materials and Methods

### Bacterial Strains, Media, and Growth Conditions

All bacterial cultures were grown in sterile LB Miller broth (Thermo Fisher Scientific, Waltham, MA, United States) at 37°C and shaking at 90 rpm. Electrocompetent XL1-Blue MRF’ *E. coli* cells (Agilent, Santa Clara, CA, United States) were used to transform the initial vector constructs. Final co-transformations and subsequent production of both the bioluminescent fusion protein (NLuc-LTF) and the control protein (NLuc) utilized chemically competent One Shot™ BL21(DE3) *E. coli* cells (Thermo Fisher Scientific). Plasmids were maintained within DE3 cells using selective pressure from the addition of ampicillin (50 μg/mL) and streptomycin (50 μg/mL). The ECOR Reference Library was obtained from the Ochman Lab (Ochman and Selander, 1984) at the University of Texas (EC990774–EC990845) and deposited in our strain collection as NRG-0125 to NRG-0196. Strain information and raw contigs from whole genome sequencing (WGS) were deposited as BioSamples SAMN13109282 to SAMN13116764 in NCBI BioProject PRJNA579348. Negative control strain, *E. coli* JW2203 (CGSC 9781), an OmpC-deficient K-12 derivative, was individually purchased from the original Keio collection (Baba et al., 2006) (Yale University, New Haven, CT, United States). Positive control strain, wild-type *E. coli* K-12 (ATCC PTA 7555), wild-type coliphage T4 (ATCC 11303-B4), and T4 propagation strain, *E. coli* DH5α (ATCC 68233) were all purchased from ATCC (ATCC, Manassas, VA, United States). A complete list of bacterial strains and their respective uses can be found in Supplementary Table 1.

### Phage T4 Propagation, Host Range Analysis, and Efficiency of Plating Calculations

Wild-type T4 was propagated in *E. coli* DH5α using standard methods. Enumeration of the propagated T4 stock was performed using the double-agar spot test method, as previously described (Clokie, 2009). Briefly, 5 mL of sterile 0.8% LB top agar (Thermo Fisher Scientific) was heated to 55°C before adding 200 μL of an *E. coli* DH5α overnight culture. This mixture was then poured onto plates of fresh LB Miller bottom agar (1.4% agar) and allowed to solidify. The propagated T4 was serially diluted in nuclease-free water (IDT, Coralville, IA, United States) under sterile conditions, and 10 μL of each dilution was spotted in triplicate onto the solidified LB plates. After drying, plates were incubated at 37°C for 18–24 h. Plaque counts were used to determine the concentration of phages (PFU/mL). Propagated phages were stored away from sunlight in phosphate buffered saline (PBS) at 4°C for up to 3 months.

Evaluations of T4-susceptibility on the negative control strain (*E. coli* JW2203), propagation strain (*E. coli* DH5α), positive control strain (*E. coli* K-12), and all 72 ECOR strains were performed in triplicate using both the double-agar spot test method and the standard full plate plaque assay method. Spot assay results were used to determine which phage dilution factor was best for each of the 72 ECOR strains, such that the chosen dilution would produce countable plaques that could be used in calculating T4’s EOP. Spot assay evaluations were also used to check for instances of lysis from without; a phenomenon that could be detected if T4 had produced fully cleared spots for early dilutions but produced no clearings and no individual plaques when at further dilutions. By comparing the results of T4’s spot assay evaluations against the ECOR library to that of the corresponding full plate plaque assays, we were also able to check for differences in the clarity of formed spots and individual plaques across the ECOR library. EOP evaluations (Clokie, 2009) for all bacterial strains relied on the resulting data from full plate plaque assays, and calculations specific were achieved by dividing the average titer of T4 on each tested ECOR strain by that of the average T4 titer on reference strain, *E. coli* DH5α. Resulting EOP values were ranked such that values >0.5 were considered “High EOP,” 0.1–0.49 were “Medium EOP,” <0.1 was “Low EOP” (Khan Mirzaei and Nilsson, 2015). ECOR strains unable to produce plaques were considered non-permissive to T4.

### Construction of NanoLuc Luciferase and NLuc-LTF Expression Vectors

The plasmids used for the expression of the luciferase-tagged T4 long tail fiber binding tip (NLuc-LTF) were: (i) pET(Ap)g57: containing the gene for required tail fiber trimerization chaperone (gp57), and (ii) pCDF(Sm)g37g38: containing genes coding for the T4 binding tip (gp37) and the additional long tail fiber chaperone gene (g38) required for the binding tip’s unique folding pattern (Galan Bartual et al., 2010). To modify T4’s long tail fiber binding tip such that it was fused to an upstream luciferase, pCDF(Sm)g37g38 was engineered to include the gene for NanoLuc Luciferase (*nluc*) as a fusion to the tail fiber binding tip. Briefly, polymerase chain reaction (PCR) was used to linearize pCDF(Sm)g37g38 just upstream of the g37 gene. Q5 Hot Start 2× Master Mix (New England Biolabs, Ipswich, MA, United States) was used for all PCR reactions, including the isolation of the codon-optimized reporter enzyme, NanoLuc Luciferase (NLuc) (Hall et al., 2012) from our previously engineered phage, NRGp4 (T7 encoding NLuc-CBM) (Hinkley et al., 2018).

Two separate versions of NLuc were produced in this study, although both were cloned immediately downstream of the N-terminal histidine tag. One version of the reporter gene was amplified to exclude the endogenous stop codon, ultimately allowing for translational fusion to T4 distal long tail fiber gene (*g37*) resulting in fusion protein, “NLuc-LTF.” The second version contained a double stop codon at the end of the *nluc* gene which prevented translational fusions and instead produced an unfused “NLuc” which was used as a control. Regions of homology that were 10 bases long on each side were added to the linearized pCDF(Sm)g37g38 and both variations of *nluc* (with and without a double stop codon), respectively, by using PCR with primer overhangs. Assembly was accomplished using NEBuilder HiFi DNA Assembly Master Mix (New England Biolabs) and final recombined constructs (“pCDF.NL_g37.g38” and “pCDF.NL”) were then respectively, transformed into electrocompetent XL1-Blue MRF’ *E. coli* cells following manufacturer recommendations. Finally, all relevant plasmids were isolated from single bacterial colonies and sequenced (Cornell Institute of Biotechnology, Core Facilities, Ithaca, NY, United States) to ensure proper assembly. A complete list of primers can be found in Supplementary Table 2, while plasmid maps are in Supplementary Figure 1.

### Expression, Isolation, and Purification of Monomeric NanoLuc Luciferase and NLuc-LTF Fusion Proteins

For expression of the binding tip fusion proteins, expression vector pCDF.NL_g37.g38 was co-transformed into BL21(DE3) *E. coli* cells alongside chaperone-producing plasmid, pET(Ap)g57. Similarly, control plasmid pCDF.NL and pET(Ap)g57 were also co-transformed into *E. coli* BL21(DE3). To induce expression of NLuc and NLuc-LTF, 300 mL of respective transformants from single colonies were grown at 37°C in LB broth supplemented with ampicillin (50 μg/mL) and streptomycin (50 μg/mL) and shaking at 90 rpm until an optical density (OD_600_) of 0.70 was attained. To slow bacterial growth and prevent inclusion bodies from forming due to protein overexpression, culture flasks were then placed in a freezer for 10 min before returning to room temperature. Once flasks were brought to room temperature, protein expression was induced by addition of isopropyl-β-D-thiogalactoside (IPTG) to a final concentration of 0.1 mM. Cultures were allowed to express proteins for 18 h under rigorous agitation (200 rpm) at 16°C. Cells were then centrifuged for 20 min at 3,260× *g* (10°C) to form a pellet. Supernatants were discarded and bacterial cells in the pellets were chemically lysed using B-PER I Bacterial Protein Extraction Reagent (Thermo Fisher Scientific) according to manufacturer guidelines. Briefly, pelleted cells underwent three freeze/thaw cycles using dry ice and hot water (75°C) exposure to facilitate lysis. Resulting crude lysates were further purified by passing them through sterile, 0.22 μm, polyvinylidene fluoride (PVDF) syringe filter units (Millipore Sigma, Burlington, MA, United States) to remove larger cellular debris.

Target proteins were purified using HisPur Cobalt Resin (Thermo Fisher Scientific) to target the N-terminal histidine repeat (6 × His) upstream of the *nluc* coding region. Extracted proteins were eluted with buffer (50 mM sodium phosphate, 300 mM sodium chloride, and 150 mM imidazole; pH 7.4), concentrated using Amicon^®^ Ultra-15 Centrifugal Filter Units (Millipore Sigma), and dialyzed using PBS with 0.15% w/v Tween 20 (Fisher BioReagents, Loughborough, United Kingdom) before being brought to the final volume of 1.5 mL. To remove protein aggregates, isolates were centrifuged at 21,000× *g* (10°C) for 2 h and stored at 4°C in 1.5 mL Protein LoBind Tubes (Eppendorf, Hamburg, Germany). Protein concentration was measured in triplicate using the Pierce™ BCA Protein Assay kit (Thermo Fisher Scientific) in clear 96-well microplates and compared to Bovine Serum Albumin (BSA) standards (Thermo Fisher Scientific). The microplate was incubated statically at 37°C for 35 min before being evaluated using a Synergy Neo2 Hybrid Multi-Mode Reader plate reader (BioTek, Winooski, VT, United States). The purity of each final protein extraction was evaluated by SDS-PAGE, using an 8 and 15% acrylamide gel to evaluate the purity of NLuc-LTF or NLuc, respectively, and comparing the 6 μL of respective denatured protein samples to 6 μL of the BenchMark Protein Ladder (Thermo Fisher Scientific). SDS-PAGE gels were run at 117V for 1.5 h using 1× SDS-PAGE Running Buffer from Bio-Rad (Bio-Rad, Hercules, CA, United States) stained with Coomassie, destained in deionized water for 2 h, and finally allowed to further destain in fresh deionized water for further band development overnight (18–20 h) before evaluation.

### Optimization of Filtration-Based Bioluminescent Adsorption Assay

**FIGURE 1:**
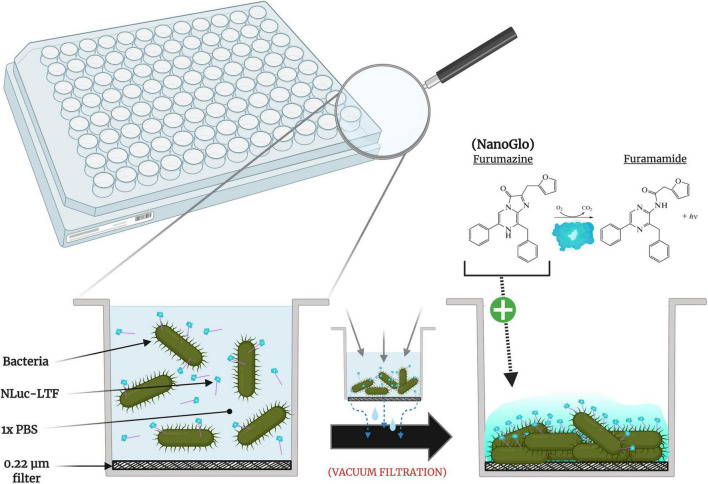
Cross-sectional view of NLuc-LTF sample well in a 96-well plate. With gp37-facilitated adsorption occurring C-terminally, N-terminal NanoLuc is free to interact with added NanoGlo substrate to produce bioluminescence.

A key component of our adsorption assay was the use of vacuum filtration applied directly on samples within 96-well filter plates (Millipore Sigma). The filters allow passage of unbound tail fibers, while the retentate consists of the bacterial cells with surface-bound tail fibers (see Figure 1). Microplates were UV-sterilized for 1 h and stored in a dry and air-tight container until day-of use. Plates were blocked for 1 h by flooding all relevant wells with 3% w/v BSA (Thermo Fisher Scientific) in PBS under mild agitation (35 rpm). The working volume of microplate wells was 15 to 350 μL, therefore wells were blocked using volumes of 360 μL. Blocking volumes and all three subsequent PBS washes were removed using a Multi-Well Plate Vacuum Manifold (PALL, Port Washington, NY, United States) and receiver plate (PALL). Final blocked microplates were stored in sterile, sealed bags at 4°C for no more than 4 h before use.

To emulate normal T4 infection conditions, log-phase cultures were used to ensure cells were metabolically active. The optimal concentration of bacteria within a volume of 200 μL was evaluated by comparing resulting filtration rates from a variety of OD_600_ values (data not shown) using both K-12 and JW2203 aliquots. Standard plate counts were performed for all 74 bacterial strains to confirm that the concentration of bacteria, or “Colony Forming Units (CFU),” was consistent between all evaluated samples at the given optical density. Adjustments to the OD_600_ were made, if needed, for individual strains to maintain consistent concentrations for testing. The NanoLuc’s substrate reagent, NanoGlo (Promega, Madison, WI, United States), was tested in triplicate against all evaluated strains to check for signs of background luminescence that could influence the assay’s signal:noise ratio.

Protein aggregation readily occurred within 24 h of normal sample storage for both NLuc and NLuc-LTF. Before the start of each experimental evaluation, isolated protein samples were centrifuged at 21,000× *g* for 2 h (10°C) and total protein content was reassessed. The optimal concentration of protein within a 50 μL volume was determined for both NLuc and NLuc-LTF and measured at a gain of 120. To equilibrate enzyme availability between NLuc and NLuc-LTF aliquots—necessary because NanoLuc constitutes only 16% of a single NLuc-LTF’s total molecular weight—respective free NLuc concentration evaluations correlated to 16% that of each evaluated NLuc-LTF aliquot (1 μg, 500 ng, 200 ng, 100 ng, and 10 ng). For example, if evaluating 200 ng of NLuc-LTF, 32 ng of NLuc would respectively, be evaluated. Due to the intensity of NanoLuc’s luminescent output, loss or gain of Relative Luminescent Unit (RLU) due to neighboring/untreated wells amplifying or muting signals was evaluated by measuring luminescence from these wells before and after the addition of NanoGlo. The effect of crossover signal between samples directly adjacent or separated by empty wells was also tested. Lastly, varying numbers of PBS washes (×3, ×4, ×5, ×6, and ×7) performed after sample incubation were evaluated to determine the optimal.

### Evaluating Adsorptive Capabilities of NanoLuc Luciferase and NLuc-LTF *via* Filtration-Based Adsorption Assay

**FIGURE 2:**
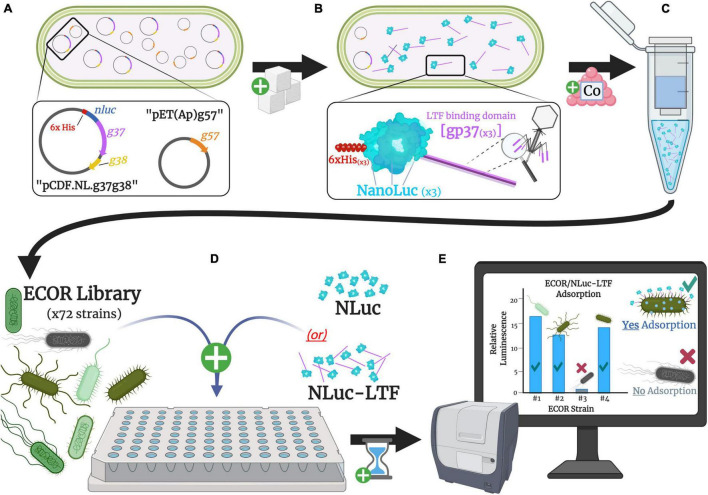
Graphical representation of experimental design. **(A)** A dual-plasmid expression system produced T4’s general trimerization chaperone (gp57A), LTF-specific chaperone (gp38), and bioluminescent fusion protein (NLuc-LTF). **(B)** Proteins were produced in *Escherichia coli* DE3 cells using 0.1 mM IPTG, before being **(C)** isolated and purified by targeting N-terminal Histidine tags with a cobalt resin. **(D)** Respective sample wells were loaded with 200 μL of a bacterial strain plus 200 ng of NLuc-LTF, incubated at room temperature for 30 min, vacuum-filtered to trap bacterial cells using 0.22 μm filters at the bottom of each well, and washed three times with phosphate buffered saline (PBS) to remove unbound proteins. **(E)** Thirty microliters of NanoGlo substrate was added to relevant wells, and bioluminescent output was evaluated in a plate reader 20–30 min later.

A graphical representation of our adsorption assay’s developed method flow is shown in Figure 2. ECOR strains and negative control strain *E. coli* JW2203 were freshly grown to the optimized OD_600_ of ∼0.50 before pelleting 2 mL of all respective strains (10,000× *g*, 22°C, 3 min), decanting the resulting supernatants, and resuspending with 2 mL of PBS. As described in the previous section, prior to use, isolated protein samples (NLuc or NLuc-LTF) were centrifuged at high speed and protein concentration was reevaluated before creating aliquots for day-of experimental use. An example of the filter plate’s experimental and optimization layouts can be found in Supplementary Figure 2.

Sample wells were loaded with 200 μL of corresponding bacterial strains and 50 μL of NLuc-LTF (200 ng) was subsequently added. Mixtures were incubated at 22°C for 30 min, vacuum-filtered, and washed three times using 360 μL of PBS to flush unbound NLuc/NLuc-LTF. Finally, 30 μL of freshly prepared NanoGlo was added directly to filters and allowed to react with bound NLuc-LTF for 20 min before measuring luminescence at a gain of 120. The limit of detection (LOD) for each evaluated microplate was also established by using the mean output of luminescence from each respective microplate’s negative control wells containing JW2203 and NLuc-LTF, so that tested samples with mean RLU outputs equal to or less than that of JW2203/NLuc-LTF’s signal were categorized as “No Adsorption Detected.” To normalize plate-to-plate variation, the mean RLU of an ECOR strain was divided by that of an on-plate control’s mean RLU (JW2203/NLuc-LTF). All samples were tested using the same batch of proteins and were all within a single week.

### Bioinformatics Analyses for T4 Genome, Expression Vectors, and ECOR Reference Library

The T4 phage genome was isolated using the manufacturer-suggested extended protocol from the Norgen Biotek Phage Genome Isolation kit (Norgen Biotek, Thorold, ON, Canada). Genome isolation for each strain of the ECOR Library was accomplished using the DNeasy Blood and Tissue DNA extraction kit (Qiagen, Hilden, Germany) and then prepared for WGS. WGS analysis was performed as a service (Animal Health Diagnostic Center, College of Veterinary Medicine, Cornell University, Ithaca, NY, United States), using the Illumina MiSeq NGS platform, a Nextera XT DNA Library Kit, and data collection with QC analysis performed in BaseSpace Sequence Hub (Illumina, San Diego, CA, United States). Raw sequence reads were assembled and analyzed using the Geneious Prime 2020.0.3 software^1^. Sanger Sequencing was performed (Biotechnology Resource Center, Cornell University, Ithaca, NY, United States) to confirm proper assembly of all constructed plasmids (Supplementary Table 2). For all relevant T4 genes (*g37*, *g38*, and *g57*), alignments between our assembled T4 genome and NCBI’s published T4 genome (NC_000866) were performed at both the nucleotide and amino acid level. For WGS, all returned contigs were evaluated to ensure the quality of the reads were >85%. T4 genome was assembled against the annotated T4 genome from NCBI. Contigs from each ECOR strain were trimmed, filtered, normalized, and error-corrected before undergoing *de novo* assembly using *E. coli* K-12 substr. MG1655 (GenBank: U00096.2) as a reference. The raw contigs from sequenced ECOR strains have been deposited in a NCBI BioProject (#PRJNA579348) for open access by future researchers. Amino acid sequences of OmpC porin proteins from *E. coli* K-12, *E. coli* O157:H7 (GenBank: BA000007.2), and *E. coli* B (GenBank: CP000819.1) were used as reference strains to align assembled OmpC sequences from the assembled ECOR strains. The “EMBOSS Protein” plug-in allowed secondary structures to be predicted for comparisons between all ECOR strain OmpC sequences.

### Statistical Analysis

Each microplate contained up to 10 ECOR strains alongside respective *E. coli* JW2203 control samples and reported resulting bioluminescent signals as RLUs. GraphPad version 8.0.0 (GraphPad Software, San Diego, CA, United States) was used to perform a Student’s *t*-test (*p*-value = 0.05, one-tailed, two samples of unequal variance) on datasets from both optimization assays and NLuc-LTF treated ECOR strain evaluations: comparing RLU outputs between treated strains and their corresponding microplate’s control samples of either “JW2203/NLuc” (optimization assay) or “JW2203/NLuc-LTF” (actual experimentation), respectively. Using a Student’s *t*-test to evaluate the difference of mean RLU output between a treated ECOR strain and that of its respective microplate’s JW2203/NLuc-LTF control allowed for binary definitions of either “yes adsorption” (*p*-value < 0.05) or “no adsorption” (*p*-value > 0.05) having occurred. All assay samples were evaluated as analytical triplicates, averaged, and reported alongside corresponding standard deviations. RLU outputs from each strain were normalized by their corresponding plate’s *E. coli* JW2203/NLuc-LTF RLU output before ranking the relative adsorptive strength of gp37 across the ECOR library.

## Results

### Host Range Analysis and Efficiency of Plating Calculations

**FIGURE 3:**
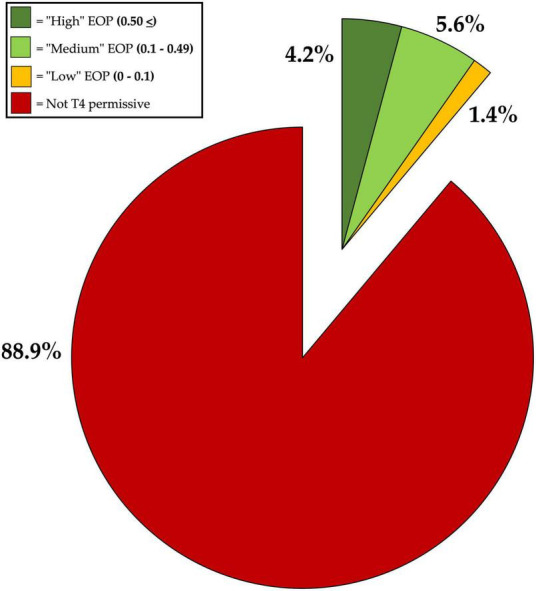
Ranked efficiency of plating (EOP) evaluations for phage T4 against *Escherichia coli* reference collection (ECOR) Library, using *E coli* DH5α as a reference strain.

The concentration of T4 was 5.5 × 10^11^ PFU/mL. Standard plaque assays showed that T4 was able to infect *E. coli* K-12 and ECOR strains #10, #13, #16, #42, #56, #60, #70, and #71. No plaques formed on the remaining 89% of the ECOR library, nor on *E. coli* JW2203; which lacks the T4 primary adsorption receptor, OmpC (Wilson et al., 1970; Montag et al., 1990; Heller, 1992). No instances of lysis from without were discovered from spot assay evaluations of T4 against the ECOR library, and comparisons between the results of spot assay and full plate plaque assays showed that there were no differences between these two datasets: including no differences found concerning clarity for either full spots or individual plaques (data not shown). Ranked EOP evaluations on susceptible ECOR strains (Figure 3) showed that #16, #70, and #71 have “High EOP” values (>0.50), #13, #42, #56, and #60 have “Medium EOP” values (0.10–0.49), and ECOR #10 had a “Low EOP” designation (<0.1). Notably, for ECOR strains #70 and #71, T4 produced higher EOP values than that of reference strain, *E. coli* DH5α. All EOP values can be found in Supplementary Table 3.

### Expression, Isolation, and Purification of Monomeric NanoLuc Luciferase and NLuc-LTF Fusion Proteins

In this study, chaperone proteins gp38 and gp57 were co-expressed during production of NLuc-LTF and NLuc. These chaperones were only needed for the trimerization and proper folding of NLuc-LTF yet were also expressed as a control measure when producing NLuc. The average yield of purified proteins was determined to be 270 and 10 μg/mL for NLuc-LTF and NLuc, respectively. A single molecule of trimeric NLuc-LTF was estimated to be 385.8 kDa in size, while a NLuc-LTF monomer was calculated to be 128.6 kDa in size: a value close to the 130 kDa band noted in lane 6 of the respective evaluated SDS-PAGE gel (Supplementary Figure 3A). Monomeric NanoLuc (NLuc) is 19.1 kDa in size, which was confirmed during SDS-PAGE analysis (Supplementary Figure 3B).

### Optimization of Filtration-Based Bioluminescent Adsorption Assay

Optimization of the filter plate assay was performed by using both wild-type *E. coli* K-12 and *E. coli* K-12_Δ_*_*ompC*_* (“JW2203”) to represent positive and negative controls for NLuc-LTF adsorption. We evaluated the maximum bacterial concentration that each well’s 0.22 μm filter could handle without subsequent fouling during the multiple rounds of washes and vacuum-filtration. To ensure that final sample volumes were never too close to the maximum working volume of 350 μL, a volume of 200 μL/well was elected for all evaluated bacterial strains. The rate of filtration when using 200 μL of a bacterial culture was evaluated across a range of optical densities (0.10, 0.30, 0.60, 0.70, and 1.00) measured at 600 nm. The OD_600_ of 0.60 allowed for the highest concentration of bacterial cells while preventing significant fouling of the filter. Plate counts performed for all 72 ECOR strains, *E. coli* K-12, and *E. coli* JW2203 at an OD_600_ of 0.60 resulted in calculated CFU/mL values that were all within a log of each another; suggesting that similar concentrations of each bacterial strain were being evaluated regardless of which strain was in use (10^8^ CFU/mL or 200 μL = 2 × 10^7^ CFU).

“Overflow” signals were produced when using 500 ng of NLuc-LTF. Therefore, 200 ng per 50 μL aliquots was used for subsequent experiments. 200 ng of NLuc-LTF correlated to using 32 ng (16% of 200 ng) of free NLuc during respective optimization evaluations (Supplementary Figure 4). Adsorption studies using K-12 and JW2203 with NLuc suggested that, in the event of NLuc-LTF adsorption to a bacterial strain’s surface the majority of the resulting adsorptive signal is most likely a product of specific LTF binding and is not significantly influenced by non-specific electrostatic charges at work between the fused upstream NanoLuc and the bacterial cell surface (Zhou and Pang, 2018). The specific placement of samples on microplates produces no statistically relevant differences, however, significant crosstalk was found to occur when wells containing different treated bacterial strains were placed directly adjacent another. To reduce crosstalk, plate maps were designed with empty wells between variants. Evaluations of washing steps showed that after three washes, no significant reduction in signal was found. Therefore, three washing steps was decided upon for subsequent experiments. Optimization data can be found within the Supplementary Figure 2.

### Evaluating Adsorptive Capabilities of NLuc-LTF and NanoLuc Luciferase *via* Filtration-Based Adsorption Assay

While phage T4 was only able to infect 8 of the 72 ECOR strains (#10, #13, #16, #42, #56, #60, #70, and #71) such that a plaque was formed, NLuc-LTF fibers were able to adsorb to all but 11 ECOR strains (#4, #5, #6, #23, #24, #25, #26, #27, #28, #46, and #47) when compared to the controls. T4 therefore has a broader “adsorptive host range” (84.7% of ECOR Library) and a more narrow “replicative host range” (11.1% of ECOR Library). This is an important distinction to be able to make when attempting to create large datasets that solely represent the step of phage adsorption.

**FIGURE 4:**
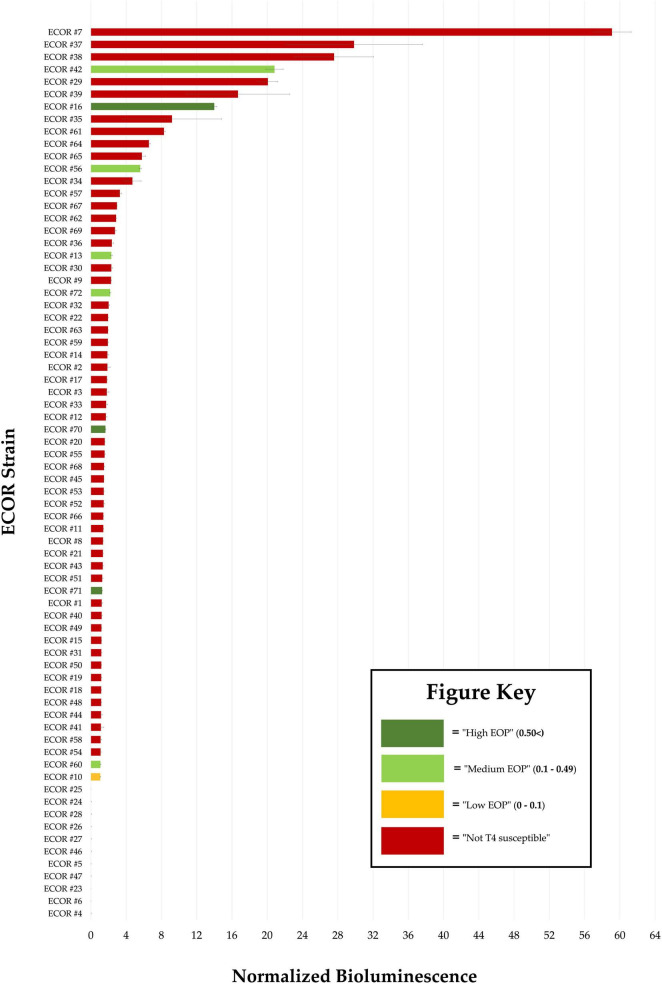
Normalized and ranked bioluminescent output of “NLuc-LTF”-treated ECOR strains. The average bioluminescent output (measured in RLU) for each treated ECOR strain was normalized by dividing the mean Relative Luminescent Unit (RLU) of an ECOR strain by that of an on-plate control’s mean RLU (JW2203). Error bars represent the standard deviation of three normalized replicates. Efficiency of plating (EOP) calculations utilized the results of T4’s full plate plaque assay evaluations. Bar colors represent (red) non-permissive T4 strains, (dark green) permissive hosts with “High EOP” (0.5<) values, (light green) permissive hosts with “Medium EOP” (0.1–0.49) values, and (yellow) permissive hosts with “Low EOP” (<0.1) values.

Individual well RLU values (Supplementary Table 4) and normalized RLU values alongside their corresponding standard deviations (Supplementary Table 5) can be found in the Supplementary Material. Student’s *t*-test evaluations utilized individual RLU outputs from each strain’s corresponding replicate wells, but normalized RLU values were used when ranking the relative adsorptive strength of NLuc-LTF against all ECOR strains as either “High,” “Medium,” or “Low” relative adsorptive strength (Figure 4). ECOR #4, #5, #6, #23, #24, #25, #26, #27, #28, #46, and #47 produced normalized RLU values that were <1 RLU and were considered to have “No Adsorption Detected (NAD),” as dictated by this assay’s determined LOD. For most ECOR strains, NLuc-LTF affinity fell within the category of “Low” adsorptive strength; defined by having a normalized RLU output between 1 and 10 normalized RLU. Three strains (#16, #29, and #39) were found to be of “Medium” adsorptive strength (normalized RLU values between 10 and 20), while strength of NLuc-LTF adsorption to ECOR #7, #37, #38, and #42 was categorized as being “High” (>20 normalized RLU).

## Discussion

For all phages, surface adsorption to a permissive bacterial host is the critical first step toward a successful phage infection. For tailed phages, initial recognition of a permissive host is facilitated by specific interactions between phage tail fibers or tail spike proteins and targeted bacterial surface receptors (Letarov and Kulikov, 2017). While not all tailed phages are the same, the modularity for both the genetic and morphologic elements commonly found in phages of the *Caudovirales* order (Veesler and Cambillau, 2011) allows our adsorption assay to be adapted for similar evaluations of other tailed phages. It is also feasible that other adsorption-specific phage proteins could be examined using our methodological design. In our proof of concept, we used coliphage T4 as our model organism and chose to evaluate T4’s long tail fiber adhesins (gp37) against the 72 isolates of the ECOR Reference Library. The ability to examine individual interactions between a singular set of a phage’s adsorptive machinery (i.e., long tail fibers, short tail fibers, tail spikes, etc.) and that protein’s targeted surface receptor was not previously possible if using common adsorption assays or classic spot and full plate plaque assays; an issue our adsorption assay is able to overcome. Being that T4 represents the most well-known of all phages, a great deal of information already exists concerning its adsorptive capabilities and replicative functions (Wilson et al., 1970; Washizaki et al., 2016; Hyman and van Raaij, 2018; Kutter et al., 2018; Duong et al., 2020b) and made T4’s adsorption machinery ideal for testing our assay.

Amino acid residues 907–996 (Washizaki et al., 2016) of T4 gp37’s distal binding tip (DT) are responsible for dictating T4’s reversible binding and initial host range specificity (Trojet et al., 2011). While random Brownian motion is responsible for initial contact between a phage and bacterium (Storms and Sauvageau, 2015), for coliphage T4, glycine islands concentrated at the tips of its LTFs allow for rapid association and disassociation with the bacterial cell as T4 “walks” across its host’s surface in search of dense enough concentrations of OmpC protein (Hyman and van Raaij, 2018). Gp37 interacts with OmpC porin proteins *via* a combination of electrostatic charges (Montag et al., 1990; Islam et al., 2019) and subsequent “lock-and-key” interactions facilitated by the globular structure (Hyman and van Raaij, 2018) of T4’s long tail fiber DT which further stabilizes adsorption as the DT fits snuggly within the actual pore formed at the center of three OmpC β-barrel structures (Galan Bartual et al., 2010). By binding at least three neighboring OmpC receptors, T4 can properly orient itself and initiate the deployment of a second set of shorter tail fibers (STFs) which interact with heptose sugars within the lipopolysaccharides’s (LPS) inner core region, ultimately resulting in tail contraction and viral genome injection (Washizaki et al., 2016; Yap et al., 2016; Islam et al., 2019).

T4’s LTFs are unique in that they can adsorb to their targeted host bacterium *via* two independent modes of interaction: OmpC-dependent or OmpC-independent (Prehm et al., 1976; Yu and Mizushima, 1982; Montag et al., 1990). Although OmpC is the preferred target receptor, if no suitable OmpC is available for T4 adsorption, gp37 can instead target exposed terminal glucose molecules within the outer core of its host’s LPS (OmpC-independent). Such is the case when T4 infects *E. coli* B strains: where OmpC proteins differ from that of *E. coli* K-12 only by the complete omission of loop 1 (see Supplementary Figure 5). In total, there are five different LPS core types: R1, R2, R3, R4, and K12 (Amor et al., 2000). Each core type has a different combination of inner and outer core structures; each with different availabilities of terminal sugars, even within the same categorization of core type. Because of gp37’s dual-receptor recognition capabilities, results from evaluations of NLuc-LTF against the ECOR library cannot be solely correlated to a specific sequence or structure of an ECOR strain’s respective OmpC or LPS core type. This would not likely be an issue for other tailed phages that tend to target a single surface receptor, as is the case, for example, with most T4-like phages (Trojet et al., 2011).

T4 was only able to form a plaque on 8 of the 72 ECOR strains (11%) due to either an inability to adsorb to the bacterial cell surface or from having been thwarted during another step of phage replication. On the other hand, evaluations of NLuc-LTF surface adsorption reflected successful binding to 61 of the 72 ECOR strains (85%). Thus, it can be said that T4’s LTF has a broader “adsorptive host range” (85%) and T4 has a more narrow “replicative host range” (11%). By overlaying these two datasets our group identified a subset of ECOR strains incompatible with T4 at the level of adsorption (ECOR #4, #5, #6, #23, #24, #25, #26, #27, #28, #46, and #47). This interpretation was only possible, however, because of the combination of using both profiling assays: ours as well as traditional plaque assays. Another interesting discovery was how, although NLuc-LTF’s adsorptive affinity was strongest toward ECOR #7, this strain is not considered to be a permissive T4 host due to T4’s inability to form a plaque. This points to the likelihood that T4 replication within ECOR #7 is likely halted at a step of replication that is post-adsorption. On the other hand, while ECOR strains #70 and #71 were infected by wild-type T4 with a “High EOP” assignment, the relative adsorptive strength of NLuc-LTF was characterized as being “Low” for both strains. This discrepancy suggests that the efficiency of T4 infection for ECOR #70 and #71 is not due to the strength of LTF adsorption but is a result of superiority at some other step in T4 infection.

Most evaluation techniques for studying phage adsorption are almost identical to the original methods created over a century ago (Sharp, 2001; Kutter and Sulakvelidze, 2004; Clokie, 2009; Clokie and Kropinski, 2009). Unfortunately, none of these available techniques can examine individual interactions between a singular set of a phage’s adsorptive machinery (i.e., long tail fibers, short tail fibers, tail spikes, etc.) and its’ targeted bacterial receptor, as they require the use of complete phages. In the context of T4, for example, the data generated from classic methods that utilize the whole phage particle would be a blur of LTF and STF interactions with a host bacterium’s surface receptors. Further, for many tailed phages when too many virions bind in tandem to a single bacterial cell this can cause depolarization of the bacterial cell membrane and result in “virion-induced lysis from without”: a process in which the bacterial cell prematurely collapses, no progeny phage are produced, no plaque is formed, and the resulting interpretation of said phage-host interaction is described as being “non-permissive” (Abedon, 2011). This would not be a problem within our evaluation assay due to phage tail contraction never occurring. While all adsorption-specific machinery must work together to facilitate proper adsorption such that genome translocation can subsequently occur, it is also important to examine the nature of phage adsorption through the individual contribution of each isolated adsorptive protein: especially because one set of a phage’s adsorption machinery may be more relevant to human medicine than the others. Consider, for example, how T4’s long tail fibers have yet to find medicinal use while its short tail fibers have been heavily investigated for their ability to strongly counteract LPS-induced inflammation *in vivo* (Miernikiewicz et al., 2016). This is not to say that current phage adsorption evaluations should be abandoned. On the contrary, especially if paired with our high-throughput bacterial binding assay, many pre-existing methodologies can generate datasets that are more in-depth than the datasets produced by either evaluation on its own. By creating an assay in which researchers can focus on a single set of a phage’s adsorptive proteins against panels of relevant bacteria, large adsorption-specific datasets can now be assembled to produce more accurate machine learning models that can eventually empower bottom-up phage engineering techniques. After all, research that aims to deepen our understanding of phage-host interactions provides the key foundation from which all future technological developments within phage-based tools will derive.

## Data Availability Statement

The original contributions presented in the study are included in the article/Supplementary Material, further inquiries can be directed to the corresponding author.

## Author Contributions

EF and SN performed the project conceptualization, methodology design, funding acquisition, and project administration. EF, AL, and TR performed the experimental investigations. EF, EP, AL, TR, BW, and SN analyzed the data. EF performed the data curation, formal analysis, and preparation of this manuscript. EF, SN, and BW undertook the experimental supervision. EF, AL, EP, TR, BW, and SN performed the manuscript reviews and edits. All authors contributed to the article and approved the submitted version.

## Conflict of Interest

The authors declare that the research was conducted in the absence of any commercial or financial relationships that could be construed as a potential conflict of interest.

## Publisher’s Note

All claims expressed in this article are solely those of the authors and do not necessarily represent those of their affiliated organizations, or those of the publisher, the editors and the reviewers. Any product that may be evaluated in this article, or claim that may be made by its manufacturer, is not guaranteed or endorsed by the publisher.

## Footnotes

1
https://www.geneious.com
