# Supplementary material for: Evaluating Phage Tail Fiber Receptor-Binding Proteins Using a Luminescent Flow-Through 96-Well Plate Assay
Source: Front Microbiol. 2021 Dec 16;12:741304. doi: 10.3389/fmicb.2021.741304 (PMC8719110; doi:10.3389/fmicb.2021.741304)
Supplement: Supplementary file 1 [file Data_Sheet_1.PDF]

Supplementary Figure 1:
Plasmid Maps

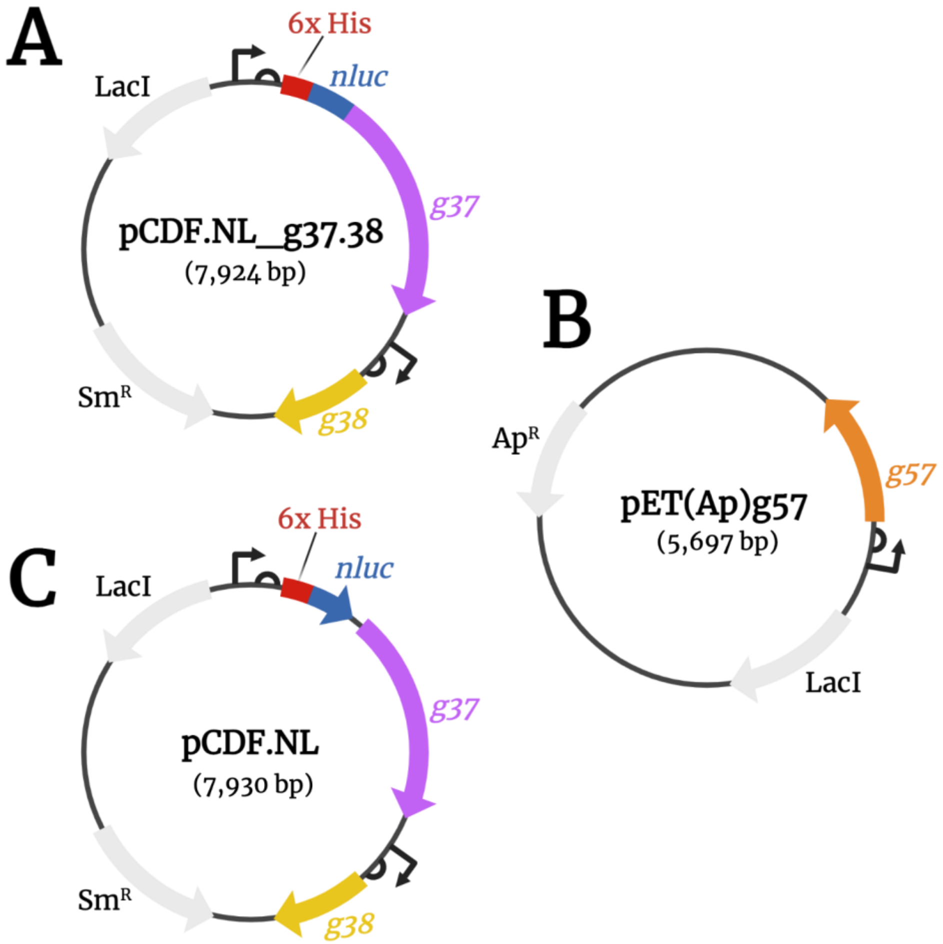

Figure S1. Expression vector constructs. **(A)** pCDF.NL_g37.g38 was used to express the NanoLuc-T4 binding tip fusion protein and LTF chaperone protein, gp38, **(B)** pET(Ap)g57 was used to express the general trimerization chaperone, gp57, and **(C)** pCDF.NL was used to express NanoLuc Luciferase only, which was used as a control.
