# Supplementary material for: Evaluating Phage Tail Fiber Receptor-Binding Proteins Using a Luminescent Flow-Through 96-Well Plate Assay
Source: Front Microbiol. 2021 Dec 16;12:741304. doi: 10.3389/fmicb.2021.741304 (PMC8719110; doi:10.3389/fmicb.2021.741304)
Supplement: Supplementary file 2 [file Data_Sheet_2.PDF]

Supplementary Figure 2:
Experimental and Optimization Microplate Set-Ups

Experimental Microplate Set-Up

	1	2	3	4	5	6	7	8	9	10	11	12
**A**	JW2203 +Nluc-LTF		Nluc-LTF alone		ECOR **A** +Nluc-LTF		ECOR **B** +Nluc-LTF		ECOR **C** +Nluc-LTF		ECOR **D** +Nluc-LTF	
**B**	JW2203 +Nluc-LTF		Nluc-LTF alone		ECOR **A** +Nluc-LTF		ECOR **B** +Nluc-LTF		ECOR **C** +Nluc-LTF		ECOR **D** +Nluc-LTF	
**C**	JW2203 +Nluc-LTF		Nluc-LTF alone		ECOR **C** +Nluc-LTF		ECOR **B** +Nluc-LTF		ECOR **C** +Nluc-LTF		ECOR **D** +Nluc-LTF	
**D**												
**E**												
**F**	ECOR **E** +Nluc-LTF		ECOR **F** +Nluc-LTF		ECOR **G** +Nluc-LTF		ECOR **H** +Nluc-LTF		ECOR **I** +Nluc-LTF		ECOR **J** +Nluc-LTF	
**G**	ECOR **E** +Nluc-LTF		ECOR **F** +Nluc-LTF		ECOR **G** +Nluc-LTF		ECOR **H** +Nluc-LTF		ECOR **I** +Nluc-LTF		ECOR **J** +Nluc-LTF	
**H**	ECOR **E** +Nluc-LTF		ECOR **F** +Nluc-LTF		ECOR **G** +Nluc-LTF		ECOR **H** +Nluc-LTF		ECOR **I** +Nluc-LTF		ECOR **J** +Nluc-LTF	

(grey, crossed)	=	Empty Well
(shaded)	=	Assay Control

*Optimization Microplate **1** of **2**.*

*	1	2	3	4	5	6	7	8	9	10	11	12
**A**	**JW2203** *(Cells only)* *((x3 washes))* X		**JW2203** +Nluc-LTF *(x3 washes)* X X X X	**JW2203** +NanoLuc* *(x3 washes)* X		**JW2203** +Nluc-LTF *(x 4 washes)* X		**JW2203** +Nluc-LTF *(x5 washes)* X		**JW2203** +Nluc-LTF *(x6 washes)* X	**JW2203** +Nluc-LTF *(x6 washes)* X	**JW2203** +Nluc-LTF *(x6 washes)* X
**B**	**JW2203** *(Cells only)* *((x3 washes))* X		**JW2203** +Nluc-LTF *(x3 washes)* X X X X	**JW2203** +NanoLuc* *(x3 washes)* X		**JW2203** +Nluc-LTF *(x 4 washes)* X		**JW2203** +Nluc-LTF *(x5 washes)* X				
**C**	**JW2203** *(Cells only)* *((x3 washes))* X		**JW2203** +Nluc-LTF *(x3 washes)* X X X X	**JW2203** +NanoLuc* *(x3 washes)* X		**JW2203** +Nluc-LTF *(x 4 washes)* X		**JW2203** +Nluc-LTF *(x5 washes)* X		**JW2203** +Nluc-LTF *(x7 washes)* X	**JW2203** +Nluc-LTF *(x7 washes)* X	**JW2203** +Nluc-LTF *(x7 washes)* X
**D**		**JW2203** +Nluc-LTF *(x3 washes)* X X X			**K-12** +NanoLuc* *(x3 washes)* X		**JW2203** +Nluc-LTF *(x3 washes)* X X X		**JW2203** +Nluc-LTF *(x3 washes)* X X X			
**E**	**K-12** *(Cells only)* X		**K-12** +Nluc-LTF *(x3 washes)* X X X X		**K-12** +NanoLuc* *(x3 washes)* X					**K-12** +Nluc-LTF *(x6 washes)* X	**K-12** +Nluc-LTF *(x6 washes)* X	**K-12** +Nluc-LTF *(x6 washes)* X
**F**	**K-12** *(Cells only)* X		**K-12** +Nluc-LTF *(x3 washes)* X X X X		**K-12** +NanoLuc* *(x3 washes)* X		**K-12** +Nluc-LTF *(x3 washes)* X X X		**K-12** +Nluc-LTF *(x3 washes)* X X X			
**G**	**K-12** *(Cells only)* X		**K-12** +Nluc-LTF *(x3 washes)* X X X X					**K-12** +Nluc-LTF *(x5 washes)* X		**K-12** +Nluc-LTF *(x7 washes)* X	**K-12** +Nluc-LTF *(x7 washes)* X	**K-12** +Nluc-LTF *(x7 washes)* X
**H**		**K-12** +Nluc-LTF *(x3 washes)* X X X		**K-12** +Nluc-LTF *(x4 washes)* X	**K-12** +Nluc-LTF *(x4 washes)* X	**K-12** +Nluc-LTF *(x4 washes)* X		**K-12** +Nluc-LTF *(x5 washes)* X	**K-12** +Nluc-LTF *(x5 washes)* X			

** = 200ng of "Nluc-LTF" was used for all relevant wells*

** = 32ng of "NanoLuc" was used for all relevant wells*

Microplate* Key

X	=	*Proves that the bacterial strains are not endogenously luminescent.*
X	=	*Proves that the placement of samples at different sections of the microplate, does not matter.*
X	=	*Proves that "NanoLuc Luciferase" is not non-specifically binding to bacterial strains and that any resulting RLU output is due to specific interactions between gp37 and the bacterial cell surface.*
X	=	*Proves that there is no statistical difference between using x3 washes or x4, x5, x6, or x7 washes. (Washes done with 1x PBS)*
	=	*Empty wells.*
	=	*Controls using negative-adsorption E. coli strain JW2203.*
	=	*Controls using positive-adsorption E. coli strain K-12.*

Optimization Microplate 2 of 2.

* *	1	2	3	4	5	6	7	8	9	10	11	12
**A**	**JW2203** *(Cells only)* X		**JW2203** +Nluc-LTF (*10 ng*) X		**JW2203** +Nluc-LTF (*100 ng*) X		**JW2203** +Nluc-LTF (*200 ng*) X		**JW2203** +Nluc-LTF (*500 ng*) X		**JW2203** +Nluc-LTF (*1000 ng*) X	
**B**	**JW2203** *(Cells only)* X		**JW2203** +Nluc-LTF (*10 ng*) X		**JW2203** +Nluc-LTF (*100 ng*) X		**JW2203** +Nluc-LTF (*200 ng*) X		**JW2203** +Nluc-LTF (*500 ng*) X		**JW2203** +Nluc-LTF (*1000 ng*) X	
**C**	**JW2203** *(Cells only)* X		**JW2203** +Nluc-LTF (*10 ng*) X		**JW2203** +Nluc-LTF (*100 ng*) X		**JW2203** +Nluc-LTF (*200 ng*) X		**JW2203** +Nluc-LTF (*500 ng*) X		**JW2203** +Nluc-LTF (*1000 ng*) X	
**D**												
**E**												
**F**	**K-12** *(Cells only)* X		**K-12** +Nluc-LTF (*10ng*) X		**K-12** +Nluc-LTF (*100ng*) X		**K-12** +Nluc-LTF (*200ng*) X		**K-12** +Nluc-LTF (*500ng*) X		**K-12** +Nluc-LTF (*1000ng*) X	
**G**	**K-12** *(Cells only)* X		**K-12** +Nluc-LTF (*10ng*) X		**K-12** +Nluc-LTF (*100ng*) X		**K-12** +Nluc-LTF (*200ng*) X		**K-12** +Nluc-LTF (*500ng*) X		**K-12** +Nluc-LTF (*1000ng*) X	
**H**	**K-12** *(Cells only)* X		**K-12** +Nluc-LTF (*10ng*) X		**K-12** +Nluc-LTF (*100ng*) X		**K-12** +Nluc-LTF (*200ng*) X		**K-12** +Nluc-LTF (*500ng*) X		**K-12** +Nluc-LTF (*1000ng*) X	

* * = *x3 washes were used for all relevant wells*

Microplate** Key

X	=	*Comparisons needed to optimize the best concentration of proteins to use during the experiment.*
(grey crossed box)	=	*Empty wells.*
(peach)	=	*Controls using negative-adsorption E. coli strain JW2203.*
(light green)	=	*Controls using positive-adsorption E. coli strain K-12.*
