# Supplementary material for: Evaluating Phage Tail Fiber Receptor-Binding Proteins Using a Luminescent Flow-Through 96-Well Plate Assay
Source: Front Microbiol. 2021 Dec 16;12:741304. doi: 10.3389/fmicb.2021.741304 (PMC8719110; doi:10.3389/fmicb.2021.741304)
Supplement: Supplementary file 3 [file Data_Sheet_3.PDF]

Supplementary Figure 3:
SDS-PAGE Analysis for fusion protein (NLuc-LTF) and control protein (NLuc)

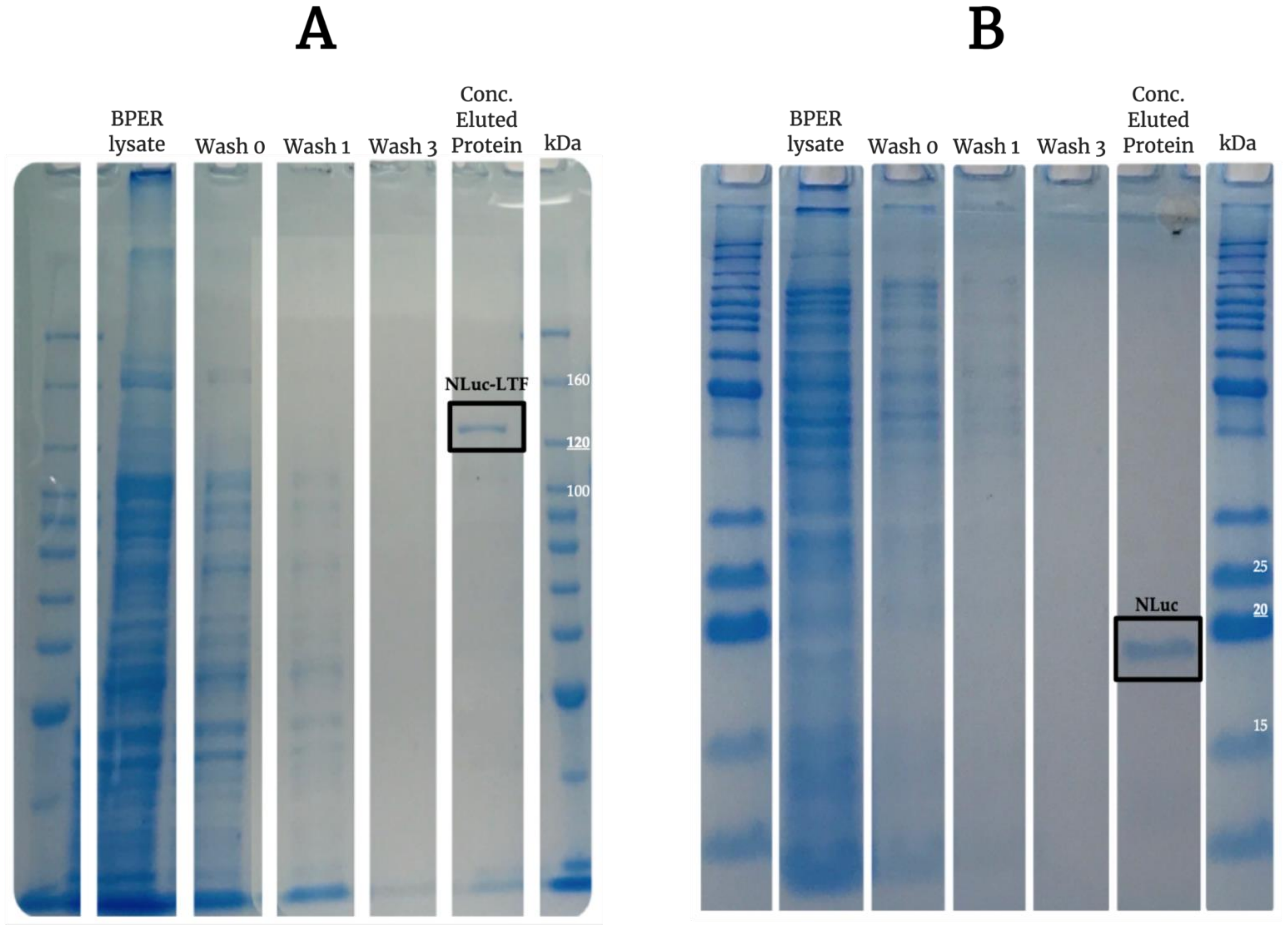

Figure S3. SDS-PAGE analysis for **(A)** fusion protein, NLuc-LTF, and **(B)** control protein, NLuc. "BPER lysate" refers to the lysate isolated at the end of the BPER I chemical digestion of induced *E. coli* cells. "Wash 0" refers to the supernatant isolated after the 1 hour of incubation between cell lysate and activated HisPure Cobalt resin. "Wash 1" and "Wash 3" refer to the first and last resin washes, respectively. "Conc. Eluted Protein" refers to the final 1.5 mL volume samples collected after dialysis in 1× PBS (0.15% Tween20). For reference of protein size, the first and last wells of each gel contained 6 µL of Thermo Fisher's BenchMark Protein Ladder.
