# Supplementary material for: Evaluating Phage Tail Fiber Receptor-Binding Proteins Using a Luminescent Flow-Through 96-Well Plate Assay
Source: Front Microbiol. 2021 Dec 16;12:741304. doi: 10.3389/fmicb.2021.741304 (PMC8719110; doi:10.3389/fmicb.2021.741304)
Supplement: Supplementary file 4 [file Data_Sheet_4.PDF]

Supplementary Figure 4:

<u>Mathematical Calculations for NLuc's Assay Concentration</u>

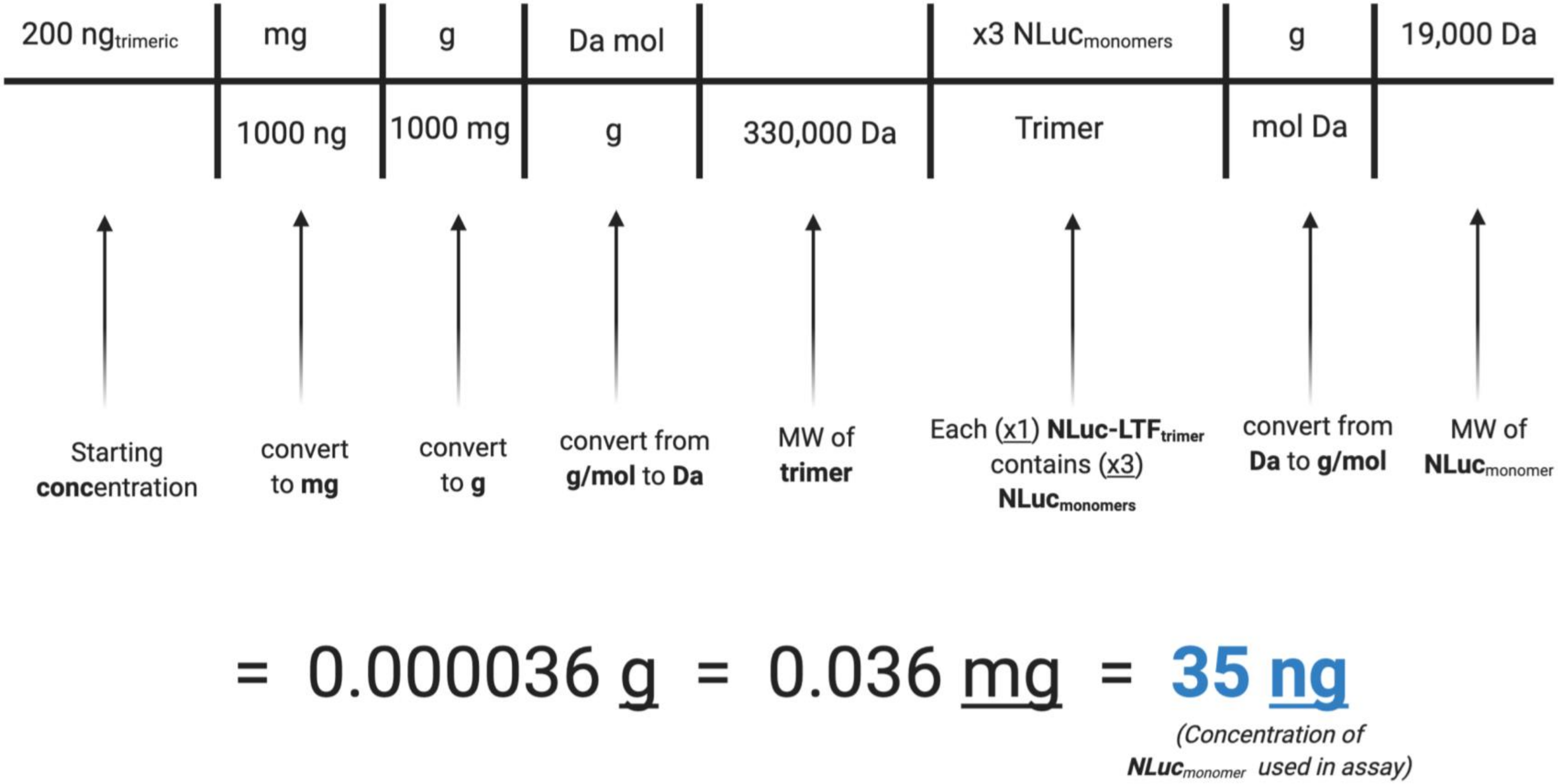

Figure S4. Mathematical calculations for NLuc's assay concentration. To equilibrate enzyme availability between NLuc and NLuc-LTF aliquots – necessary because NanoLuc constitutes only 16% of a single NLuc-LTF's total molecular weight – respective free NLuc concentration evaluations correlated to 16% that of each evaluated NLuc-LTF aliquot (1 µg, 500 ng, 200 ng, 100 ng, and 10 ng). For example, if evaluating 200 ng of NLuc-LTF, 32 ng of NLuc would respectively be evaluated.
