# Supplementary material for: Evaluating Phage Tail Fiber Receptor-Binding Proteins Using a Luminescent Flow-Through 96-Well Plate Assay
Source: Front Microbiol. 2021 Dec 16;12:741304. doi: 10.3389/fmicb.2021.741304 (PMC8719110; doi:10.3389/fmicb.2021.741304)
Supplement: Supplementary file 5 [file Data_Sheet_5.PDF]

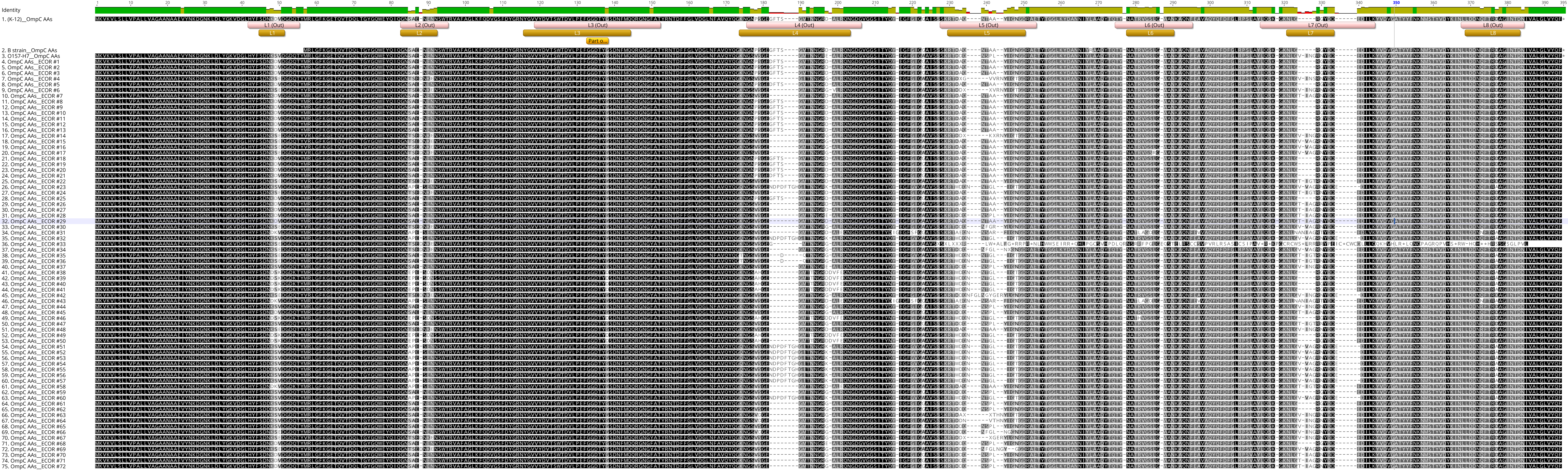
Identity
1. (K-12)__OmpC AAs
L1 (Out)
L2 (Out)
L3 (Out)
L4 (Out)
L5 (Out)
L6 (Out)
L7 (Out)
L8 (Out)
L1
L2
L3
L4
L5
L6
L7
L8
Part o...
2. B strain__OmpC AAs
3. O157-H7__OmpC AAs
4. OmpC AAs__ECOR #1
5. OmpC AAs__ECOR #2
6. OmpC AAs__ECOR #3
7. OmpC AAs__ECOR #4
8. OmpC AAs__ECOR #5
9. OmpC AAs__ECOR #6
10. OmpC AAs__ECOR #7
11. OmpC AAs__ECOR #8
12. OmpC AAs__ECOR #9
13. OmpC AAs__ECOR #10
14. OmpC AAs__ECOR #11
15. OmpC AAs__ECOR #12
16. OmpC AAs__ECOR #13
17. OmpC AAs__ECOR #14
18. OmpC AAs__ECOR #15
19. OmpC AAs__ECOR #16
20. OmpC AAs__ECOR #17
21. OmpC AAs__ECOR #18
22. OmpC AAs__ECOR #19
23. OmpC AAs__ECOR #20
24. OmpC AAs__ECOR #21
25. OmpC AAs__ECOR #22
26. OmpC AAs__ECOR #23
27. OmpC AAs__ECOR #24
28. OmpC AAs__ECOR #25
29. OmpC AAs__ECOR #26
30. OmpC AAs__ECOR #27
31. OmpC AAs__ECOR #28
32. OmpC AAs__ECOR #29
33. OmpC AAs__ECOR #30
34. OmpC AAs__ECOR #31
35. OmpC AAs__ECOR #32
36. OmpC AAs__ECOR #33
37. OmpC AAs__ECOR #34
38. OmpC AAs__ECOR #35
39. OmpC AAs__ECOR #36
40. OmpC AAs__ECOR #37
41. OmpC AAs__ECOR #38
42. OmpC AAs__ECOR #39
43. OmpC AAs__ECOR #40
44. OmpC AAs__ECOR #41
45. OmpC AAs__ECOR #42
46. OmpC AAs__ECOR #43
47. OmpC AAs__ECOR #44
48. OmpC AAs__ECOR #45
49. OmpC AAs__ECOR #46
50. OmpC AAs__ECOR #47
51. OmpC AAs__ECOR #48
52. OmpC AAs__ECOR #49
53. OmpC AAs__ECOR #50
54. OmpC AAs__ECOR #51
55. OmpC AAs__ECOR #52
56. OmpC AAs__ECOR #53
57. OmpC AAs__ECOR #54
58. OmpC AAs__ECOR #55
59. OmpC AAs__ECOR #56
60. OmpC AAs__ECOR #57
61. OmpC AAs__ECOR #58
62. OmpC AAs__ECOR #59
63. OmpC AAs__ECOR #60
64. OmpC AAs__ECOR #61
65. OmpC AAs__ECOR #62
66. OmpC AAs__ECOR #63
67. OmpC AAs__ECOR #64
68. OmpC AAs__ECOR #65
69. OmpC AAs__ECOR #66
70. OmpC AAs__ECOR #67
71. OmpC AAs__ECOR #68
72. OmpC AAs__ECOR #69
73. OmpC AAs__ECOR #70
74. OmpC AAs__ECOR #71
75. OmpC AAs__ECOR #72
