# Supplementary material for: Evaluating Phage Tail Fiber Receptor-Binding Proteins Using a Luminescent Flow-Through 96-Well Plate Assay
Source: Front Microbiol. 2021 Dec 16;12:741304. doi: 10.3389/fmicb.2021.741304 (PMC8719110; doi:10.3389/fmicb.2021.741304)
Supplement: Supplementary file 6 [file Data_Sheet_6.PDF]

Supplementary Table 1:
Bacterial strains, bacteriophages, and plasmids

Bacteria Name	Experimental Use	Source
E. coli K12	Positive control strain for assay optimization.	ATCC
E. coli DH5*a*	Host for $T4_{wt}$ propagation and reference strain for E.O.P. calculations. *E. coli* K-12 derivative.	ATCC
E. coli JW2203	Negative control strain used in assay optimization and present on each tested filter plate.	Yale University
Electrocompetent XL1 Blue-MRF'	Used to transform constructed expression plasmids and ensure stability of construct.	Agilent
One Shot™ BL21 (DE3*) E. coli* cells	Used to co-transform and subsequently express relevant chaperone proteins and corresponding expression vectors.	Invitrogen/ThermoScientific
ECOR Reference Library (x72 Strains)	Panel of 72 representative *E. coli* strains used to determine effectiveness of our Bioluminescent Adsorption Assay.	University of Texas

Bacteriophage Name	Experimental Use	Source
$T4_{wt}$	Whole genome sequencing, host range analysis, and E.O.P. calculations.	ATCC

Plasmid Name	Experimental Use	Source
pCDF-Duet	N/A	Used as the foundation for Dr. Van Raaij's donated "pCDF(Sm)g37g38" T4 long tail fiber expression vector.
pET21a(+)	N/A	Used as the foundation for Dr. Van Raaij's donated "pET(Ap)g57" T4 chaperone-expression vector.
pCDF(Sm)g37g38	PCR linearized to insert codon optimized NanoLuc Luciferase (NLuc).	Donated by Dr. Van Raaij.
pET(Ap)g57	Used "as is" to express T4's required trimerization chaperone, gp57.	Donated by Dr. Van Raaij.
pCDF.NL_g37.g38	Engineered expression vector which produces bioluminescent fusion protein (NLuc-LTF) and corresponding gp37-specific chaperone protein (gp38).	Engineered in-house.
pCDF.NLmono	Engineered expression vector which produces bioluminescent control protein (NLuc).	Engineered in-house.

Table S1. List of the bacterial strains, bacteriophages, and plasmids used in this study.
