# Supplementary material for: Evaluating Phage Tail Fiber Receptor-Binding Proteins Using a Luminescent Flow-Through 96-Well Plate Assay
Source: Front Microbiol. 2021 Dec 16;12:741304. doi: 10.3389/fmicb.2021.741304 (PMC8719110; doi:10.3389/fmicb.2021.741304)
Supplement: Supplementary file 7 [file Data_Sheet_7.PDF]

Supplementary Table 2:

<u>Primers Used for Sequencing and Plasmid Design</u>

**<u>KEY</u>**		
**lower-case letters**	-->	*ROHs (Regions Of Homology)*
**UPPER-CASE letters**	-->	*Annealing portion of the primer*
**<u>Underlined</u> letters**	-->	*Start or Stop codon utilized as coding region*

Primers used in <u>plasmid construction</u>:

Amplicon #	Amplicon Name	(For. / Rev.) Primer #s	(5' - 3') Primer Sequences	Relevant Features	Template
#1	NanoLuc-V1	#1827 / #1828	(F) ATGGTATTCACACTGGAGG<br><br>(R) CTGCTAAAATGCGTTCAC	No stop codon present. Intended to transcriptionally fuse with downstream tail fiber genes. Start codon present, but true coding region begins farther upstream in order to include the 6xHis tag.	"T7(Nluc)"…....aka......" pNRG-004"
#2	ROH/ NanoLuc-V1	#1927 / #1928	(F) caccacagccagATGGTATTCACACTGGAG<br><br>(R) tgcttggatcCTGCTAAAATGCGTTCAC	Regions of homology (ROH) corresponding to PCR-linearized pCDF(Sm)g37g38 were added to both the 5' and 3' end of the amplicon. Intended to be used in homologous recombination in order to produce a vector capable of producing NanoLuc/gp37 fusion proteins.	Amplicon **#1**
#3	NanoLuc-V2	#1827 / #1832	(F) ATGGTATTCACACTGGAGG<br><br>(R) C<u>TTATTA</u>TGCTAAAATGCGTTCACAA	Contains a double stop codon. Intended to end NanoLuc's coding region and produce monomeric NanoLuc Luciferase. Start codon present, but true coding region begins farther upstream in order to include the 6xHis tag.	"T7(Nluc)"…........."pNRG-004"
#4	ROH/ NanoLuc-V2	#1927 / #1932	(F) caccacagccagATGGTATTCACACTGGAG<br><br>(R) tgcttggatcC<u>TTATTA</u>TGCTAAAATGCGTTCACAA	Regions of homology (ROH) corresponding to PCR-linearized pCDF(Sm)g37g38 were added to both the 5' and 3' end of the amplicon. Intended to be used in homologous recombination to create a vector capable of producing monomeric NanoLuc proteins.	Amplicon **#3**

#5	*PCR-linearized,* pCDF(Sm)g37g38	#1923 / #1924	(F) GATCCAAGCAAAATCGCAG<br><br>(R) CTGGCTGTGGTGATGATG	Gifted plasmid from Van Raaij Lab is linearized such that the immediate 5' is the beginning of gp37's coding region. End of the coding region is the endogenous stop codon of gp37. Intended to be used in creation of expression vectors.	"pCDF(Sm)g37g38"
#6	ROH-V1/ *PCR-linearized,* pCDF(Sm)g37g38	#1925 / #1926	(F) cattttagcaGATCCAAGCAAAATCGCAG<br><br>(R) tgaataccatCTGGCTGTGGTGATGATG	Regions of homology (ROH) corresponding NanoLuc-V1 were added to both the 5' and 3' end of the amplicon. Intended to be used in homologous recombination with NanoLuc-V1 in order to produce a vector capable of producing NanoLuc/gp37 fusion proteins.	Amplicon **#5**
#7	ROH-V2/ *PCR-linearized,* pCDF(Sm)g37g38	#1929 / #1926	(F) agcataataaGATCCAAGCAAAATCGCAG<br><br>(R) tgaataccatCTGGCTGTGGTGATGATG	Regions of homology (ROH) corresponding NanoLuc-V2 were added to both the 5' and 3' end of the amplicon. Intended to be used in homologous recombination with NanoLuc-V2 in order to produce a vector capable of producing monomeric NanoLuc proteins.	

Primers used for Sequencing:

Amplicon #	Amplicon Name	(For. / Rev.) Primer #s	(5' - 3') Primer Sequences	Template
#8	**"pCDF*" Variants (***Sequencing Fragment***)**	#868 / #941	(F) CACGGTAAAACGCCAC<br><br>(R) GCTAGTTATTGCTCAGCGG	pCDF(Sm)g37g38…...**OR**…...pCDF.NL_g37.g38…….**OR…...**pCDF.Nluc-mono
#9	"**pET(Ap)g57**"s (*Sequencing Fragment*)	N/A	(F) CGATTTAGAGCTTGACG<br><br>(R) CAGTAGTAGGTTGAGGC	

Table S2. List of primers used for Sequencing or plasmid design.
