# Supplementary material for: Evaluating Phage Tail Fiber Receptor-Binding Proteins Using a Luminescent Flow-Through 96-Well Plate Assay
Source: Front Microbiol. 2021 Dec 16;12:741304. doi: 10.3389/fmicb.2021.741304 (PMC8719110; doi:10.3389/fmicb.2021.741304)
Supplement: Supplementary file 8 [file Data_Sheet_8.PDF]

Supplementary Table 3:

T4 Host Range and E.O.P. Calculations

**Bacteria**	**(wavelength = 600nm) Avg. Optical Density of ECOR Strains, JW2203, and K-12 *Plate Counts***	**(CFU/mL) Bacterial Concentration from ECOR Strains, JW2203, and K-12 *Plate Counts***	**(wavelength = 600nm) Average Optical Density of *Tested ECOR Samples******	**(wavelength = 600nm) Average (Day-specific) Optical Density of JW2203 from relevant *Tested ECOR Samples******	**E.O.P. Calculated from Avg'd Dilution Sets**	**E.O.P. Rankng**
ECOR #1	0.59	1.90E+08	0.50	0.50		
ECOR #2	0.61	2.40E+08	0.51	0.50		
ECOR #3	0.58	2.60E+08	0.52	0.50		
ECOR #4	0.64	2.03E+08	0.50	0.50		
ECOR #5	0.61	2.27E+08	0.50	0.50		
ECOR #6	0.58	4.67E+07	0.51	0.50		
ECOR #7	0.61	2.27E+08	0.50	0.50		
ECOR #8	0.60	5.67E+07	0.50	0.50		
ECOR #9	0.62	2.47E+08	0.50	0.50		
ECOR #10	0.60	3.20E+08	0.50	0.50	0.02	**Low E.O.P.**
ECOR #11	0.59	3.30E+08	0.51	0.50		
ECOR #12	0.62	2.47E+08	0.50	0.50		
ECOR #13	0.58	1.29E+08	0.50	0.50	0.43	**Medium E.O.P.**
ECOR #14	0.58	1.93E+08	0.52	0.50		
ECOR #15	0.61	2.80E+08	0.50	0.50		
ECOR #16	0.62	1.73E+08	0.52	0.50	0.78	**High E.O.P.**
ECOR #17	0.62	5.66E+08	0.50	0.50		
ECOR #18	0.59	2.03E+08	0.50	0.50		
ECOR #19	0.60	7.70E+08	0.50	0.50		

ECOR **#20**	0.60	2.76E+08	0.50	0.50		
ECOR **#21**	0.58	6.97E+08	0.51	0.51		
ECOR **#22**	0.65	6.93E+08	0.50	0.51		
ECOR **#23**	0.59	9.57E+08	0.52	0.51		
ECOR **#24**	0.61	5.53E+08	0.51	0.51		
ECOR **#25**	0.57	9.21E+08	0.50	0.51		
ECOR **#26**	0.58	8.41E+08	0.50	0.50		
ECOR **#27**	0.63	1.07E+08	0.50	0.50		
ECOR **#28**	0.61	2.86E+08	0.51	0.50		
ECOR **#29**	0.61	1.51E+08	0.50	0.50		
ECOR **#30**	0.57	3.02E+08	0.50	0.50		
ECOR **#31**	0.59	2.94E+08	0.50	0.54		
ECOR **#32**	0.58	5.34E+08	0.51	0.54		
ECOR **#33**	0.63	5.18E+08	0.50	0.54		
ECOR **#34**	0.63	2.04E+08	0.52	0.54		
ECOR **#35**	0.58	6.04E+08	0.50	0.54		
ECOR **#36**	0.58	6.58E+08	0.50	0.50		
ECOR **#37**	0.60	3.77E+08	0.50	0.54		
ECOR **#38**	0.60	5.29E+08	0.51	0.54		
ECOR **#39**	0.58	4.78E+08	0.54	0.54		
ECOR **#40**	0.58	4.74E+08	0.50	0.50		
ECOR **#41**	0.60	6.05E+08	0.50	0.50		
ECOR **#42**	0.58	5.52E+08	0.53	0.50	0.47	**Medium E.O.P.**
ECOR **#43**	0.58	2.01E+08	0.52	0.50		
ECOR **#44**	0.57	2.57E+08	0.51	0.50		
ECOR **#45**	0.57	6.78E+08	0.54	0.50		
ECOR **#46**	0.58	1.98E+08	0.50	0.50		
ECOR **#47**	0.58	3.15E+08	0.50	0.50		
ECOR **#48**	0.58	1.53E+08	0.52	0.50		
ECOR **#49**	0.57	2.68E+08	0.50	0.50		
ECOR **#50**	0.58	1.08E+08	0.51	0.50		

ECOR #**51**	0.59	2.14E+08	0.50	0.50		
ECOR #**52**	0.62	3.17E+08	0.52	0.51		
ECOR #**53**	0.60	2.43E+08	0.52	0.51		
ECOR #**54**	0.62	2.94E+08	0.51	0.51		
ECOR #**55**	0.57	3.50E+08	0.50	0.51		
ECOR #**56**	0.58	2.83E+08	0.52	0.51	0.36	**Medium E.O.P.**
ECOR #**57**	0.58	1.10E+08	0.52	0.51		
ECOR #**58**	0.62	2.47E+08	0.52	0.51		
ECOR #**59**	0.60	3.40E+08	0.51	0.51		
ECOR #**60**	0.62	3.17E+08	0.51	0.51	0.15	**Medium E.O.P.**
ECOR #**61**	0.58	1.07E+08	0.51	0.51		
ECOR #**62**	0.58	2.13E+08	0.52	0.51		
ECOR #**63**	0.58	1.53E+08	0.50	0.51		
ECOR #**64**	0.57	3.50E+08	0.51	0.51		
ECOR #**65**	0.58	3.13E+08	0.51	0.51		
ECOR #**66**	0.57	2.83E+08	0.52	0.51		
ECOR #**67**	0.58	1.40E+08	0.51	0.51		
ECOR #**68**	0.57	1.97E+08	0.51	0.51		
ECOR #**69**	0.62	2.70E+08	0.52	0.51		
ECOR #**70**	0.60	1.97E+08	0.51	0.51	1.40	**High E.O.P.**
ECOR #**71**	0.58	6.30E+08	0.52	0.51	3.40	**High E.O.P.**
ECOR #**72**	0.63	2.83E+08	0.50	0.51		
**JW2203**	0.52	1.65E+08	-	-	-	-
**K-12**	0.58	2.22E+08	-	-	-	-

**KEY**					
**"High E.O.P."**		=	0.50<	(ECOR #**16**, #**70**, #**71**)	4.2%
**"Medium E.O.P."**		=	0.1 - 0.49	(ECOR #**13**, #**42**, #**56**, #**60**)	5.6%
**"Low E.O.P."**		=	0 - 0.1	(ECOR #**10**)	1.4%
**"Not a host"**		=	<0.1	-	88.9%

Table S3. Plaque Assays to define T4 host range within ECOR library and subsequent EOP calculations.
