# Supplementary material for: Evaluating Phage Tail Fiber Receptor-Binding Proteins Using a Luminescent Flow-Through 96-Well Plate Assay
Source: Front Microbiol. 2021 Dec 16;12:741304. doi: 10.3389/fmicb.2021.741304 (PMC8719110; doi:10.3389/fmicb.2021.741304)
Supplement: Supplementary file 9 [file Data_Sheet_9.PDF]

Supplementary Table 4:
(RLU Outputs) Individual Samples of all ECOR/NLuc-LTF Treatments

**KEY**		
	-->	"**ECOR/Nluc-LTF**" sample has RLU values that are significantly lower than "**JW2203/Nluc-LTF**" sample values. In this instance, a *p*-value ≤ 0.05 does **NOT** correspond to an adsorption event. This
UNDERLINED/**BOLD**	-->	The calculated *p*- value is NOT statistically significant.

Table S4. RLU Outputs of individual samples wells for all ECOR/NLuc-LTF treatment strains.

ECOR Strain	T4 Plaquing Host	(Amor et al., 2000) LPS Core Type	("ECOR/NLuc-LTF") RLU Output			("ECOR/Nluc-LTF") Averaged RLU	("ECOR/Nluc-LTF") Standard Deviation	("JW2203/NLuc-LTF ") RLU Output			("JW2203/Nluc-LTF") Averaged RLU	("JW2203/Nluc-LTF") Standard Deviation	(1-tail) *p* -value "ECOR/Nluc-LTF" *vs.* "JW2203/Nluc-LTF"	Adsorptive Host? ("JW2203/Nluc-LTF" *p* -value ≤ 0.05)
ECOR #1		R2	2387	2385	2641	*2471*	147	2015	2075	1955	*2015*	60	0.01	Yes
ECOR #2		K-12	4358	3177	3768	*3768*	591	2015	2075	1955	*2015*	60	0.02	Yes
ECOR #3		K-12	3084	4193	3639	*3639*	555	2015	2075	1955	*2015*	60	0.02	Yes
ECOR #4		R4	1970	2152	1898	*2007*	131	2015	2075	1955	*2015*	60	**0.46**	---
ECOR #5		R2	1807	1854	1831	*1831*	24	2015	2075	1955	*2015*	60	0.01	---
ECOR #6		R3	10779	9941	10360	*10360*	419	10788	10167	10377	*10444*	316	**0.40**	---
ECOR #7		R1	119000	118153	119846	*119000*	847	2015	2075	1955	*2015*	60	0.00	Yes
ECOR #8		R2	2770	2921	2619	*2770*	151	2015	2075	1955	*2015*	60	0.00	Yes
ECOR #9		R3	4600	4623	4577	*4600*	23	2015	2075	1955	*2015*	60	0.00	Yes
ECOR #10	Yes.	R2	11057	11023	11091	*11057*	34	10788	10167	10377	*10444*	316	0.04	Yes
ECOR #11		R2	9097	9409	8658	*9055*	377	6707	6440	6173	*6440*	267	0.00	Yes
ECOR #12		R2	4871	4556	4714	*4714*	158	2665	2889	2777	*2777*	112	0.00	Yes
ECOR #13	Yes.	K-12	6420	6346	6493	*6420*	74	2665	2889	2777	*2777*	112	0.00	Yes
ECOR #14		K-12	5402	5000	5201	*5201*	201	2665	2889	2777	*2777*	112	0.00	Yes
ECOR #15		R1	3231	3394	3313	*3313*	82	2665	2889	2777	*2777*	112	0.00	Yes
ECOR #16	Yes.	R1	38047	39727	38887	*38887*	840	2665	2889	2777	*2777*	112	0.00	Yes
ECOR #17		R1	4968	5144	5056	*5056*	88	2665	2889	2777	*2777*	112	0.00	Yes
ECOR #18		R1	3238	3238	3237	*3238*	1	2665	2889	2777	*2777*	112	0.01	Yes
ECOR #19		R1	3044	3226	3487	*3252*	223	2665	2889	2777	*2777*	112	0.02	Yes
ECOR #20		R1	4252	4494	4373	*4373*	121	2665	2889	2777	*2777*	112	0.02	Yes
ECOR #21		R1	8963	9103	8822	*8963*	141	6625	6528	6577	*6577*	49	0.00	Yes
ECOR #22		R1	13159	12910	12360	*12810*	409	6625	6528	6577	*6577*	49	0.00	Yes
ECOR #23		R1	6521	6508	6515	*6515*	7	6625	6528	6577	*6577*	49	**0.08**	---
ECOR #24		R1	5040	4650	5526	*5072*	439	6625	6528	6577	*6577*	49	0.01	---
ECOR #25		R2	4637	4675	4713	*4675*	38	6625	6528	6577	*6577*	49	0.00	---
ECOR #26		R3	4682	4669	4610	*4654*	38	5660	5364	5961	*5662*	299	0.01	---
ECOR #27		R3	4638	4321	5465	*4808*	591	5660	5364	5961	*5662*	299	**0.06**	---
ECOR #28		R3	4682	4521	4275	*4493*	205	5660	5364	5961	*5662*	299	0.00	Yes
ECOR #29		R1	112815	114087	113451	*113451*	636	5660	5364	5961	*5662*	299	0.00	Yes
ECOR #30		R1	13068	13075	13097	*13080*	15	5660	5364	5961	*5662*	299	0.00	Yes
ECOR #31		R2	6169	6215	6260	*6215*	46	5260	4969	5551	*5260*	291	0.01	Yes
ECOR #32		R1	10546	10594	10641	*10594*	48	5260	4969	5551	*5260*	291	0.00	Yes
ECOR #33		R1	10051	8166	9111	*9109*	943	5260	4969	5551	*5260*	291	0.01	Yes
ECOR #34		R1	20455	28794	24625	*24625*	4170	5260	4969	5551	*5260*	291	0.01	Yes
ECOR #35		R1	17350	52742	77368	*49153*	30170	5260	4969	5551	*5260*	291	0.05	Yes
ECOR #36		R1	9907	9609	10204	*9907*	298	4101	4461	3982	*4181*	249	0.00	Yes
ECOR #37		R3	155513	187701	123324	*155513*	32189	5260	4969	5551	*5260*	291	0.01	Yes
ECOR #38		R1	126672	162195	144434	*144434*	17762	5260	4969	5551	*5260*	291	0.00	Yes
ECOR #39		R1	86608	112616	60600	*86608*	26008	5260	4969	5551	*5260*	291	0.02	Yes
ECOR #40		R1	5096	5082	5067	*5082*	15	4101	4461	3982	*4181*	249	0.01	Yes
ECOR #41		R1	5925	5123	4596	*5215*	669	4101	4461	3982	*4181*	249	0.05	Yes
ECOR #42	Yes.	R1	56948	56118	57778	*56948*	830	2852	2723	2639	*2738*	107	0.00	Yes
ECOR #43		R4	3681	3684	3677	*3681*	4	2852	2723	2639	*2738*	107	0.00	Yes
ECOR #44		R1	4806	4531	5081	*4806*	275	4101	4461	3982	*4181*	249	0.02	Yes
ECOR #45		R3	4174	4058	3942	*4058*	116	2852	2723	2639	*2738*	107	0.00	Yes
ECOR #46		R1	2685	2265	2475	*2475*	210	2852	2723	2639	*2738*	107	**0.07**	---
ECOR #47		R1	2768	2564	2666	*2666*	102	2852	2723	2639	*2738*	107	**0.22**	---
ECOR #48		R1	3195	3178	3187	*3187*	9	2852	2723	2639	*2738*	107	0.01	Yes
ECOR #49		R1	3281	3261	3271	*3271*	10	2852	2723	2639	*2738*	107	0.01	Yes
ECOR #50		R1	3199	3222	3245	*3222*	23	2852	2723	2639	*2738*	107	0.01	Yes
ECOR #51		R1	3927	3523	3134	*3528*	397	2852	2723	2639	*2738*	107	0.03	Yes
ECOR #52		R1	5226	5383	5540	*5383*	157	3706	3841	3571	*3706*	135	0.00	Yes
ECOR #53		R1	5416	5396	5376	*5396*	20	3706	3841	3571	*3706*	135	0.00	Yes
ECOR #54		R1	10926	11807	11615	*11449*	463	10788	10167	10377	*10444*	316	0.02	Yes
ECOR #55		R1	5755	5806	5781	*5781*	26	3706	3841	3571	*3706*	135	0.00	Yes
ECOR #56	Yes.	R1	59395	58376	57357	*58376*	1019	10788	10167	10377	*10444*	316	0.00	Yes
ECOR #57		R1	12922	12564	11110	*12199*	960	3706	3841	3571	*3706*	135	0.00	Yes
ECOR #58		R3	3827	4070	4312	*4070*	243	3706	3841	3571	*3706*	135	0.05	Yes
ECOR #59		R1	7317	7132	6946	*7132*	186	3706	3841	3571	*3706*	135	0.00	Yes
ECOR #60	Yes.	R1	11488	11506	10747	*11247*	433	10788	10167	10377	*10444*	316	**0.03**	Yes
ECOR #61		R1	30711	31283	30138	*30711*	573	3706	3841	3571	*3706*	135	0.00	Yes
ECOR #62		R1	8915	8876	8837	*8876*	39	3184	3043	3087	*3105*	72	0.00	Yes
ECOR #63		R1	6142	6028	5914	*6028*	114	3184	3043	3087	*3105*	72	0.00	Yes
ECOR #64		R1	20395	20441	20418	*20418*	23	3184	3043	3087	*3105*	72	0.00	Yes
ECOR #65		R1	17951	16581	19320	*17951*	1370	3184	3043	3087	*3105*	72	0.00	Yes
ECOR #66		R1	4557	4193	4375	*4375*	182	3184	3043	3087	*3105*	72	0.00	Yes
ECOR #67		R1	9362	8987	9175	*9175*	188	3184	3043	3087	*3105*	72	0.00	Yes
ECOR #68		R1	4520	4620	4719	*4620*	100	3184	3043	3087	*3105*	72	0.00	Yes
ECOR #69		R1	8484	8767	8166	*8472*	301	3184	3043	3087	*3105*	72	0.00	Yes
ECOR #70	Yes.	R1	17256	16894	17617	*17256*	362	10788	10167	10377	*10444*	316	0.00	Yes
ECOR #71	Yes.	R1	13136	13507	13322	*13322*	186	10788	10167	10377	*10444*	316	0.00	Yes
ECOR #72		R1	15733	14867	15300	*15300*	433	7001	7077	6925	*7001*	76	0.00	Yes
