# Supplementary material for: Evaluating Phage Tail Fiber Receptor-Binding Proteins Using a Luminescent Flow-Through 96-Well Plate Assay
Source: Front Microbiol. 2021 Dec 16;12:741304. doi: 10.3389/fmicb.2021.741304 (PMC8719110; doi:10.3389/fmicb.2021.741304)
Supplement: Supplementary file 10 [file Data_Sheet_10.PDF]

# Supplementary Table 5:
# **(Normalized RLU Outputs) All ECOR/NLuc-LTF Treatments**

| **KEY** |
|---|
| "**High**" = >20 RLU |
| "**Medium**" = 10 - 20 RLU |
| "Low" = 1 - 10 RLU |
| "*N.A.D.*" = <1 RLU |

**Table S5**. Normalized Data for all ECOR/NLuc-LTF treatments.

| ECOR Strain | ("ECOR/NLuc-LTF") Normalized Individual RLU Output | | | ("ECOR/Nluc-LTF") Standard Deviation of Normalized Individual RLU | ("ECOR/Nluc-LTF") Averaged RLU | ("JW2203/Nluc-LTF") Averaged RLU | ("ECOR/Nluc-LTF" vs. "JW2203/Nluc-LTF") Normalized RLU Averages | (High, Medium, Low, "N.A.D") (("ECOR/Nluc-LTF" vs. "JW2203/Nluc-LTF")) Adsorptive Strength |
|---|---|---|---|---|---|---|---|---|
| ECOR #1 | 1.18 | 1.15 | 1.35 | 0.11 | 2471 | 2015 | 1.23 | Low |
| ECOR #2 | 2.16 | 1.53 | 1.93 | 0.32 | 3768 | 2015 | 1.87 | Low |
| ECOR #3 | 1.53 | 2.02 | 1.86 | 0.25 | 3639 | 2015 | 1.80 | Low |
| ECOR #4 | 0.98 | 1.04 | 0.97 | 0.04 | 2007 | 2015 | 1.00 | |
| ECOR #5 | 0.90 | 0.89 | 0.94 | 0.02 | 1831 | 2015 | 0.91 | |
| ECOR #6 | 1.00 | 0.98 | 1.00 | 0.01 | 10360 | 10444 | 0.99 | |
| ECOR #7 | 59.06 | 56.94 | 61.30 | 2.18 | 119000 | 2015 | 59.10 | High |
| ECOR #8 | 1.37 | 1.41 | 1.34 | 0.03 | 2770 | 2015 | 1.37 | Low |
| ECOR #9 | 2.28 | 2.23 | 2.34 | 0.06 | 4600 | 2015 | 2.28 | Low |
| ECOR #10 | 1.02 | 1.08 | 1.07 | 0.03 | 11057 | 10444 | 1.06 | Low |
| ECOR #11 | 1.36 | 1.46 | 1.40 | 0.05 | 9055 | 6440 | 1.41 | Low |
| ECOR #12 | 1.83 | 1.58 | 1.70 | 0.13 | 4714 | 2777 | 1.70 | Low |
| ECOR #13 | 2.41 | 2.20 | 2.34 | 0.11 | 6420 | 2777 | 2.31 | Low |
| ECOR #14 | 2.03 | 1.73 | 1.87 | 0.15 | 5201 | 2777 | 1.88 | Low |
| ECOR #15 | 1.21 | 1.17 | 1.19 | 0.02 | 3313 | 2777 | 1.19 | Low |
| ECOR #16 | 14.28 | 13.75 | 14.00 | 0.26 | 38887 | 2777 | 14.01 | Medium |
| ECOR #17 | 1.86 | 1.78 | 1.82 | 0.04 | 5056 | 2777 | 1.82 | Low |
| ECOR #18 | 1.21 | 1.12 | 1.17 | 0.05 | 3238 | 2777 | 1.17 | Low |
| ECOR #19 | 1.14 | 1.12 | 1.26 | 0.07 | 3252 | 2777 | 1.17 | Low |
| ECOR #20 | 1.60 | 1.56 | 1.57 | 0.02 | 4373 | 2777 | 1.58 | Low |
| ECOR #21 | 1.35 | 1.39 | 1.34 | 0.03 | 8963 | 6577 | 1.36 | Low |
| ECOR #22 | 1.99 | 1.98 | 1.88 | 0.06 | 12810 | 6577 | 1.95 | Low |
| ECOR #23 | 0.98 | 1.00 | 0.99 | 0.01 | 6515 | 6577 | 0.99 | |
| ECOR #24 | 0.76 | 0.71 | 0.84 | 0.06 | 5072 | 6577 | 0.77 | |
| ECOR #25 | 0.70 | 0.72 | 0.72 | 0.01 | 4675 | 6577 | 0.71 | |
| ECOR #26 | 0.83 | 0.87 | 0.77 | 0.05 | 4654 | 5662 | 0.82 | |
| ECOR #27 | 0.82 | 0.81 | 0.92 | 0.06 | 4808 | 5662 | 0.85 | |
| ECOR #28 | 0.83 | 0.84 | 0.72 | 0.07 | 4493 | 5662 | 0.80 | |
| ECOR #29 | 19.93 | 21.27 | 19.03 | 1.13 | 113451 | 5662 | 20.08 | Medium |
| ECOR #30 | 2.31 | 2.44 | 2.20 | 0.12 | 13080 | 5662 | 2.31 | Low |
| ECOR #31 | 1.17 | 1.25 | 1.13 | 0.06 | 6215 | 5260 | 1.18 | Low |
| ECOR #32 | 2.00 | 2.13 | 1.92 | 0.11 | 10594 | 5260 | 2.02 | Low |
| ECOR #33 | 1.91 | 1.64 | 1.64 | 0.16 | 9109 | 5260 | 1.73 | Low |
| ECOR #34 | 3.89 | 5.79 | 4.44 | 0.98 | 24625 | 5260 | 4.71 | Low |
| ECOR #35 | 3.26 | 9.83 | 14.50 | 5.65 | 48834 | 5260 | 9.20 | Low |
| ECOR #36 | 2.42 | 2.15 | 2.56 | 0.21 | 9907 | 4181 | 2.38 | Low |
| ECOR #37 | 29.57 | 37.77 | 22.22 | 7.78 | 155513 | 5260 | 29.85 | High |
| ECOR #38 | 24.08 | 32.64 | 26.02 | 4.49 | 144434 | 5260 | 27.58 | High |
| ECOR #39 | 16.47 | 22.66 | 10.92 | 5.88 | 86608 | 5260 | 16.68 | Medium |
| ECOR #40 | 1.24 | 1.14 | 1.27 | 0.07 | 5082 | 4181 | 1.22 | Low |
| ECOR #41 | 1.42 | 1.23 | 1.12 | 0.15 | 4696 | 4181 | 1.26 | Low |
| ECOR #42 | 19.97 | 20.61 | 21.89 | 0.98 | 56948 | 2738 | 20.82 | High |
| ECOR #43 | 1.29 | 1.35 | 1.39 | 0.05 | 3681 | 2738 | 1.35 | Low |
| ECOR #44 | 1.17 | 1.02 | 1.28 | 0.13 | 4806 | 4181 | 1.15 | Low |
| ECOR #45 | 1.46 | 1.49 | 1.49 | 0.02 | 4058 | 2738 | 1.48 | Low |
| ECOR #46 | 0.94 | 0.83 | 0.94 | 0.06 | 2475 | 2738 | 0.90 | |
| ECOR #47 | 0.97 | 0.94 | 1.01 | 0.03 | 2666 | 2738 | 0.97 | |
| ECOR #48 | 1.12 | 1.17 | 1.21 | 0.04 | 3187 | 2738 | 1.16 | Low |
| ECOR #49 | 1.15 | 1.20 | 1.24 | 0.04 | 3271 | 2738 | 1.20 | Low |
| ECOR #50 | 1.12 | 1.18 | 1.23 | 0.05 | 3222 | 2738 | 1.18 | Low |
| ECOR #51 | 1.38 | 1.29 | 1.19 | 0.09 | 3528 | 2738 | 1.29 | Low |
| ECOR #52 | 1.41 | 1.40 | 1.55 | 0.08 | 5383 | 3706 | 1.45 | Low |
| ECOR #53 | 1.46 | 1.40 | 1.51 | 0.05 | 5396 | 3706 | 1.46 | Low |
| ECOR #54 | 1.01 | 1.16 | 1.12 | 0.08 | 11449 | 10444 | 1.10 | Low |
| ECOR #55 | 1.55 | 1.51 | 1.62 | 0.05 | 5781 | 3706 | 1.56 | Low |
| ECOR #56 | 5.51 | 5.74 | 5.53 | 0.13 | 58376 | 10444 | 5.59 | Low |
| ECOR #57 | 3.49 | 3.27 | 3.11 | 0.19 | 12199 | 3706 | 3.29 | Low |
| ECOR #58 | 1.03 | 1.06 | 1.21 | 0.09 | 4070 | 3706 | 1.10 | Low |
| ECOR #59 | 1.97 | 1.86 | 1.95 | 0.06 | 7132 | 3706 | 1.93 | Low |
| ECOR #60 | 1.06 | 1.13 | 1.04 | 0.05 | 11247 | 10444 | 1.08 | Low |
| ECOR #61 | 8.29 | 8.14 | 8.44 | 0.15 | 30711 | 3706 | 8.29 | Low |
| ECOR #62 | 2.80 | 2.92 | 2.86 | 0.06 | 8876 | 3105 | 2.86 | Low |
| ECOR #63 | 1.93 | 1.98 | 1.92 | 0.03 | 6028 | 3105 | 1.94 | Low |
| ECOR #64 | 6.41 | 6.72 | 6.61 | 0.16 | 20418 | 3105 | 6.58 | Low |
| ECOR #65 | 5.64 | 5.45 | 6.26 | 0.42 | 17951 | 3105 | 5.78 | Low |
| ECOR #66 | 1.43 | 1.38 | 1.42 | 0.03 | 4375 | 3105 | 1.41 | Low |
| ECOR #67 | 2.94 | 2.95 | 2.97 | 0.02 | 9175 | 3105 | 2.96 | Low |
| ECOR #68 | 1.42 | 1.52 | 1.53 | 0.06 | 4620 | 3105 | 1.49 | Low |
| ECOR #69 | 2.66 | 2.88 | 2.65 | 0.13 | 8472 | 3105 | 2.73 | Low |
| ECOR #70 | 1.60 | 1.66 | 1.70 | 0.05 | 17256 | 10444 | 1.65 | Low |
| ECOR #71 | 1.22 | 1.33 | 1.28 | 0.06 | 13322 | 10444 | 1.28 | Low |
| ECOR #72 | 2.25 | 2.10 | 2.21 | 0.08 | 15300 | 7001 | 2.19 | Low |
