# Supplementary material for: Evaluating Phage Tail Fiber Receptor-Binding Proteins Using a Luminescent Flow-Through 96-Well Plate Assay
Source: Front Microbiol. 2021 Dec 16;12:741304. doi: 10.3389/fmicb.2021.741304 (PMC8719110; doi:10.3389/fmicb.2021.741304)
Supplement: Supplementary file 11 [file Data_Sheet_11.PDF]

Supplementary Table 6:

Information Collected about ECOR Reference Library Features

ECOR Strain	T4 Forms Plaques?	(Amor et al., 2000) O:H Serotype	(Amor et al., 2000) LPS Core Type	(Amor et al., 2000) Phylogenic Group	(Boyd and Hartl, 1998) "*kps*" Type II Capsule	(Courtesy of Global Good) Refractory	(Courtesy of Global Good) Geographical Source	(Courtesy of Global Good) Biological Source	(Washizake et al., 2016) LPS Core Type Composition
ECOR #1		O144:H4	R2	A		Refractory	Iowa	Human (F)	
ECOR #2		O48:H32	K-12	A			New York	Human (M)	Main chain = **Glc I, Glc II,** and **Glc III**........Branches = **Gal** *attached to* **Glc I** and **Hep IV-GlcNAc** *attached to* **Glc III**
ECOR #3		O1:H32	K-12	A			Massachusetts	Dog	Main chain = **Glc I, Glc II,** and **Glc III**........Branches = **Gal** *attached to* **Glc I** and **Hep IV-GlcNAc** *attached to* **Glc III**
ECOR #4		OR:H?	R4	A			Iowa	Human (F)	
ECOR #5		O?:H6	R2	A			Iowa	Human (F)	
ECOR #6		O173:H?	R3	A			Iowa	Human (M)	Main chain of the outer core = **Glc I, Gal I,** and **Glc II**........Branches = **GlcNAc branch** *attached to* **Gal I** & **Glc III branch** *attached to* **Glc II**
ECOR #7		O8:H45	R1	A			Washington (zoo)	Orangutan	
ECOR #8		O86:H2	R2	A	*kps1,2*	Refractory	Iowa	Human (F)	
ECOR #9		O167:H-	R3	A			Sweeden	Human (F)	Main chain of the outer core = **Glc I, Gal I,** and **Glc II**........Branches = **GlcNAc branch** *attached to* **Gal I** & **Glc III branch** *attached to* **Glc II**
ECOR #10	Yes.	O6:H10	R2	A		Refractory	New York	Human (F)	
ECOR #11		O10:H-	R2	A	*kps1,2*		Sweeden	Human (F) Urine	
ECOR #12		O?:H32	R2	A			Sweeden	Human (F)	
ECOR #13	Yes.	OR:H25	K-12	A			Sweeden	Human (F)	Main chain = **Glc I, Glc II,** and **Glc III**........Branches = **Gal** *attached to* **Glc I** and **Hep IV-GlcNAc** *attached to* **Glc III**
ECOR #14		O72:H4	K-12	A	*kps1,2*		Sweeden	Human (F) Urine	Main chain = **Glc I, Glc II,** and **Glc III**........Branches = **Gal** *attached to* **Glc I** and **Hep IV-GlcNAc** *attached to* **Glc III**
ECOR #15		O25:H30	R1	A			Sweeden	Human (F)	
ECOR #16	Yes.	O9:H10	R1	A			Washington (zoo)	Leopard	
ECOR #17		O29:H-	R1	A		Refractory	Indonesia	Pig	
ECOR #18		O?:H11	R1	A		Refractory	Washington (zoo)	Celebese ape	
ECOR #19		O89:H?	R1	A		Refractory	Washington (zoo)	Celebese ape	
ECOR #20		O121:H11	R1	A			Bali	Steer	
ECOR #21		O121:H11	R1	A			Bali	Steer	
ECOR #22		O150:H28	R1	A			Bali	Steer	
ECOR #23		O25:H1	R1	A		Refractory	Washington (zoo)	Elephant	
ECOR #24		O15:H-	R1	A	*kps1,2*		Sweeden	Human (F)	
ECOR #25		O127:H40	R2	A			New York	Dog	
ECOR #26		O104:H21	R3	B1			Massachusetts	Human infant	Main chain of the outer core = **Glc I, Gal I,** and **Glc II**........Branches = **GlcNAc branch** *attached to* **Gal I** & **Glc III branch** *attached to* **Glc II**
ECOR #27		O104:H21	R3	B1			Washington (zoo)	Giraffe	Main chain of the outer core = **Glc I, Gal I,** and **Glc II**........Branches = **GlcNAc branch** *attached to* **Gal I** & **Glc III branch** *attached to* **Glc II**
ECOR #28		O104:H2	R3	B1			Iowa	Human (F)	Main chain of the outer core = **Glc I, Gal I,** and **Glc II**........Branches = **GlcNAc branch** *attached to* **Gal I** & **Glc III branch** *attached to* **Glc II**
ECOR #29		O150:H21	R1	B1		Refractory	Nevada	Kangaroo rat	
ECOR #30		O113:H21	R1	B1			Alberta	Bison	
ECOR #31		O79:H25	R2	E		Refractory	Washington (zoo)	Leopard	
ECOR #32		O25:H1	R1	B1			Washington (zoo)	Giraffe	
ECOR #33		O7:H21	R1	B1			California	Sheep	
ECOR #34		O88:H-	R1	B1			Massachusetts	Dog	
ECOR #35		O1:H-	R1	D	*kps1,2*	Refractory	Iowa	Human (M)	
ECOR #36		O1:H-	R1	D	*kps1,2*		Iowa	Human (F)	
ECOR #37		O55:H7	R3	E		Refractory	Washington (zoo)	Marmoset	Main chain of the outer core = **Glc I, Gal I,** and **Glc II**........Branches = **GlcNAc branch** *attached to* **Gal I** & **Glc III branch** *attached to* **Glc II**
ECOR #38		O7:H-	R1	D	*kps1,2*	Refractory	Iowa	Human (F)	
ECOR #39		O7:H-	R1	D	*kps1,2*		Sweeden	Human (F)	
ECOR #40		O7:H-	R1	D	*kps1,2*		Sweeden	Human (F) Urine	
ECOR #41		O7:H-	R1	D	*kps1,2*		Tonga	Human (M)	
ECOR #42	Yes.	O87:H26	R1	E			Massachusetts	Human (M)	
ECOR #43		O?:H18	R4	E			Sweeden	Human (F)	
ECOR #44		O17:H34	R1	D	*kps1,2*		Washington (zoo)	Cougar	
ECOR #45		O?:H2	R3				Indonesia	Pig	Main chain of the outer core = **Glc I, Gal I,** and **Glc II**........Branches = **GlcNAc branch** *attached to* **Gal I** & **Glc III branch** *attached to* **Glc II**
ECOR #46		O1:H-	R1	D	*kps1,2*		Washington (zoo)	Celebese ape	
ECOR #47		O17:H18	R1	D	*kps1,2*		New Guinea	Sheep	
ECOR #48		O23:H15	R1	D	*kps1,2*		Sweeden	Human (F) Urine	
ECOR #49		O2:H4	R1	D	*kps1,2*	Refractory	Sweeden	Human (F)	
ECOR #50		O2:H4	R1	D	*kps1,2*	Refractory	Sweeden	Human (F) Urine	
ECOR #51		O25:H1	R1	B2	*kps1,2*		Massachusetts	Human infant	
ECOR #52		O25:H1	R1	B2	*kps1,2*		Washington (zoo)	Orangutan	
ECOR #53		O4:H5	R1	B2	*kps1,2*		Iowa	Human (F)	
ECOR #54		O25:H1	R1	B2	*kps1,2*		Iowa	Human	
ECOR #55		O25:H1	R1	B2	*kps1,2*		Sweeden	Human (F)	
ECOR #56	Yes.	O6:H10	R1	B2	*kps1,2*		Sweeden	Human (F) Urine	
ECOR #57		O2:H1	R1	B2	*kps1,2*		Washington (zoo)	Gorilla	
ECOR #58		O112:H8	R3	B1			Washington (zoo)	Lion	Main chain of the outer core = **Glc I, Gal I,** and **Glc II**........Branches = **GlcNAc branch** *attached to* **Gal I** & **Glc III branch** *attached to* **Glc II**
ECOR #59		O2:H4	R1	B2			Massachusetts	Human (M)	
ECOR #60	Yes.	O4:H5	R1	B2			Sweeden	Human (F) Urine	
ECOR #61		O2:H4	R1	B2	*kps1,2*		Sweeden	Human (F)	
ECOR #62		O2:H4	R1	B2	*kps1,2*		Sweeden	Human (F) Urine	
ECOR #63		OR:H-	R1	B2	*kps1,2*		Sweeden	Human (F)	
ECOR #64		O75:H-	R1	B2	*kps1,2*		Sweeden	Human (F) Urine	
ECOR #65		O8:H10	R1	B2			Washington (zoo)	Celebese ape	
ECOR #66		O4:H40	R1	B2	*kps1,2*		Washington (zoo)	Celebese ape	
ECOR #67		O141:H49	R1	B1			Indonesia	Goat	
ECOR #68		O25:H21	R1	B1		Refractory	Washington (zoo)	Giraffe	
ECOR #69		O86:H10	R1	B1			Washington (zoo)	Celebese ape	
ECOR #70		O78:H-	R1	B1			Washington (zoo)	Gorilla	
ECOR #71	Yes.	OR:H19	R1	B1			Sweeden	Human (F) Urine	
ECOR #72		O8:H30	R1	B1			Sweeden	Human (F) Urine	
